# Pairwise maximum entropy model explains the role of white matter structure in shaping emergent co-activation states

**DOI:** 10.1038/s42003-021-01700-6

**Published:** 2021-02-16

**Authors:** Arian Ashourvan, Preya Shah, Adam Pines, Shi Gu, Christopher W. Lynn, Danielle S. Bassett, Kathryn A. Davis, Brian Litt

**Affiliations:** 1 Department of Bioengineering, School of Engineering and Applied Science, University of Pennsylvania, Philadelphia, PA USA; 2 Penn Center for Neuroengineering and Therapeutics, University of Pennsylvania, Philadelphia, PA USA; 3 Department of Computer Science and Engineering, University of Electronic Science and Technology of China, Chengdu, China; 4 Department of Physics & Astronomy, School of Arts & Sciences, University of Pennsylvania, Philadelphia, PA USA; 5 Department of Electrical and Systems Engineering, School of Engineering and Applied Science, University of Pennsylvania, Philadelphia, PA USA; 6 Department of Psychiatry, Perelman School of Medicine, University of Pennsylvania, Philadelphia, PA USA; 7 Department of Neurology, Hospital of the University of Pennsylvania, Philadelphia, PA USA

**Keywords:** Computational neuroscience, Computational models

## Abstract

A major challenge in neuroscience is determining a quantitative relationship between the brain’s white matter structural connectivity and emergent activity. We seek to uncover the intrinsic relationship among brain regions fundamental to their functional activity by constructing a pairwise maximum entropy model (MEM) of the inter-ictal activation patterns of five patients with medically refractory epilepsy over an average of ~14 hours of band-passed intracranial EEG (iEEG) recordings per patient. We find that the pairwise MEM accurately predicts iEEG electrodes’ activation patterns’ probability and their pairwise correlations. We demonstrate that the estimated pairwise MEM’s interaction weights predict structural connectivity and its strength over several frequencies significantly beyond what is expected based solely on sampled regions’ distance in most patients. Together, the pairwise MEM offers a framework for explaining iEEG functional connectivity and provides insight into how the brain’s structural connectome gives rise to large-scale activation patterns by promoting co-activation between connected structures.

## Abstract (web summary)

Ashourvan et al. apply a pairwise maximum entropy model to intracranial EEG data from epileptic patients to infer structure-function relationships. Their results indicate that the pairwise MEM model provides insight into the structural connectivity of the brain and how it gives rise to activity patterns.

## Introduction

An age-old question in neuroscience concerns the enigmatic relationship between the brain’s large-scale function and its underlying white matter structure^1–3^. How does the complex array of functional dynamics observed in the brain emerge from the static architecture of structural white matter connections (i.e., structural connectivity) between brain regions? Previous studies demonstrate that the resting-state fMRI functional connectivity networks, defined as the statistical relationships between all pairs of sampled brain regions’ time series^4^, share important organizational features with the structural connectome^5–9^. Moreover, studies have discovered hierarchical and small-world organization in both the functional connectivity between brain regions as well as in the network of structural wires connecting them^10–12^. Additionally, coupling between functional and structural connectivity is not constant over time, but rather evolves throughout adolescence^13^, and is altered in mental health disorders^14,15^. Commonly used functional connectivity measures, such as the Pearson correlation, can reveal dynamic patterns of neural activity and the brain’s functional organization at short (e.g.,^16^) and long time scales (e.g.,^17,18^), respectively.

Such model-free measures, however, quantify the functional similarity between neural units (e.g., neurons or brain regions), and therefore are fundamentally limited in their ability to discern between different underlying mechanisms^19,20^. For example, given two brain regions with a high Pearson correlation, one cannot distinguish between the following three scenarios: (i) The regions are communicating directly via a structural connection, (ii) the regions are communicating indirectly via a structural pathway bridging intermediate regions, or (iii) the two regions are not communicating at all, but are rather being driven by a common third region. Recent research shows that accounting for indirect and higher-order structural connections between brain regions can further improve predictions of the functional connectivity that is supported^21,22^.

To distinguish between direct and indirect communication, one must begin with a model of how patterns of activity are generated in the brain and then infer the network of underlying interactions that best describes correlations in the data. Biophysical microcircuit modeling such as dynamical causal modeling^23,24^ aims to address these limitations using neural mass models of synaptic dynamics, informed by empirical ion channel and structural priors. Although these biophysical models enable us to estimate the effective connectivity between a small set of brain regions, the space of parameters rapidly expands as more regions are incorporated in the model. More recently, one stochastic model of neural activity – the pairwise maximum entropy model (MEM) – has generated particular interest, primarily because it is formally minimal in the sense that it accounts for the observed pairwise correlations in the data while remaining explicitly agnostic to all higher-order correlations^25^.

In recent studies, this model has proven sensitive to the spatiotemporal co-activation patterns in neuronal spiking at the micro-scale^26–28^ and in patterns of blood-oxygen-level-dependent (BOLD) activity at the macro-scale^29–31^. The pairwise MEM is based on the principle of maximum entropy, which states that the probability distribution (i.e., the model) that best represents one’s current state of knowledge about a system is the one with the largest entropy (or uncertainty) in the context of previously observed data. Fitting a pairwise MEM entails iteratively adjusting the strength of individual region activation and all region pair interactions until the estimated correlations match the correlations observed in the data. In this way, the MEM makes quantitative predictions about the frequencies of global activity patterns, rather than simply quantifying the similarities between regions, as is common in studies of functional connectivity. Notably, prior studies using resting-state fMRI data have demonstrated that the pairwise MEM accurately predicts the observed patterns of regional activations, and provides a more accurate map of the underlying structural connectivity than conventional functional connectivity methods^29^.

As the number of sampled brain regions (*N*) increases, the number of all possible states increases exponentially (2^*N*^). Therefore, it is progressively more likely that we will not observe many of the activation states as we record from more regions. Based on this intuition, it has been suggested that for a dataset with *N* regions and *L* samples, the accuracy of the pairwise MEM scales as a function of $\frac{L}{{2}^{N}}$^32^. Consequently, these observations suggest that once the dimensionality of recordings exceeds ≈ 25 regions, brute force sampling is no longer a viable strategy for measuring the underlying distributions of all states. However, while there are 2^*N*^ possible states, there is reason to expect that a much smaller number of measurements is sufficient for capturing the fundamental structure essential to the collective behavior in the recorded regions. For instance, Tkačik et al. demonstrated that it is possible to infer maximum entropy models for more than one hundred neurons from approximately only 2 h of data without any sign of overfitting^33^. Therefore, in theory, large datasets with relatively static correlation structure would allow the pairwise MEM to estimate the expected probabilities of activation states.

In the same vein, we examine several hours (14.6 ± 2.5) of iEEG recordings over a day in 5 patients with partial-onset refractory epilepsy across several frequency bands, between 4–180 Hz, and show that the functional connectivity between brain regions are effectively static when examined over extended recordings (>12 h). We hypothesize that fitting a pairwise MEM allows us to capture the essential underlying structure that shapes the observed functional activation patterns. We construct a series of pairwise MEMs capable of accurately reproducing the correlation structure observed in binarized iEEG power amplitude states. We observe a high cross-frequency similarity between the functional connectivity matrices estimated using pairwise MEM, which suggests that a common underlying mechanism supports the functional dynamics across all frequency bands. Therefore, we hypothesize that the brain’s physical wiring likely comprises this common scaffold, and we test this hypothesis by comparing the network topology of the inferred maximum entropy interactions with the architecture of white-matter fibers between regions. We observe strong correlations between the structural connectivity and pairwise MEM interactions. Receiver operating characteristic curve analysis also reveals that the pairwise MEM provides accurate predictions of the underlying structural connectivity across several frequency bands in most patients. To control for spatial autocorrelation^34,35^, we test the significance of these findings by proposing a resampling approach for creating subject-specific structural nulls with conserved distance features. Finally, we show that the pairwise MEM explains the observed co-activation states’ probability over a broad range of frequencies. Together, these findings demonstrate the pairwise MEM’s utility in explaining the functional connectivity and co-activation patterns in multi-channel iEEG and uncovering their underlying anatomical substrates.

## Results

### Pairwise correlations stabilize over several hours of iEEG recordings

**Fig. 1:**
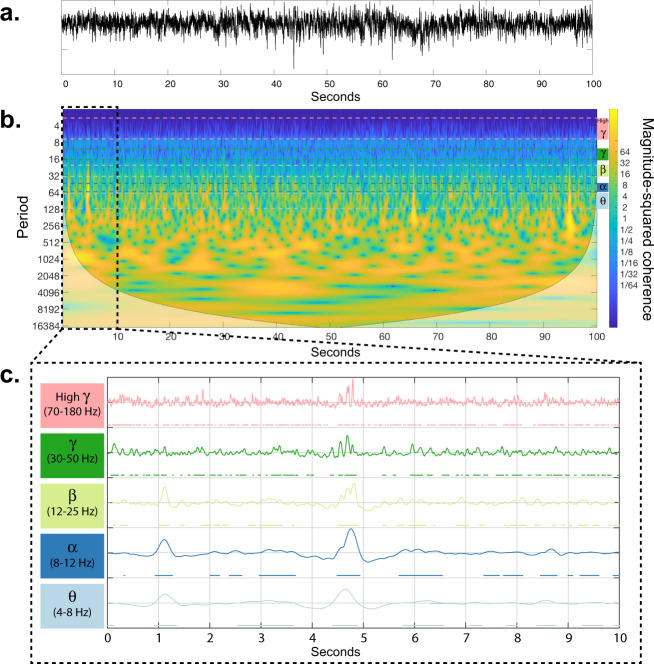
Construction of power amplitude states from iEEG time-series. **a** The raw iEEG time-series recorded from a sample electrode. **b** A wavelet decomposition of 100 s of raw iEEG time-series^81^. Color-coded dashed lines show the *θ*, *α*, *β*, *γ*, and high *γ* frequency bands. **c** The average power of all frequencies within each band is high-pass filtered at 0.5 Hz, and normalized to obtain a *z*-score. The resultant time series were then thresholded at zero separately for each band, to create the binarized power amplitude states on which the MEM operates. Note that the ‘on’ states are marked by color-coded dots under each curve.

**Fig. 2:**
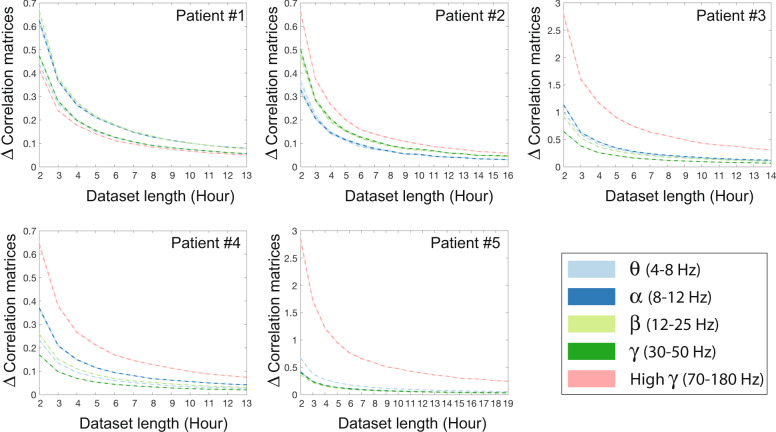
The relationship between length of dataset and stability of pairwise correlations. Plots show the mean (10,000 iterations) difference between the two correlation matrices calculated from randomly selected (1-h inter-ictal segments) datasets with lengths of *n* and *n* − 1 hours. We use the matrix norm to quantify the difference between correlations matrices. The shaded-area around the means (dashed line) highlights the standard error, and colors indicate the frequency bands.

Here, we examine the volume of data needed to establish stable relationships between brain regions. We do this by tracking the magnitude of the difference between pairwise correlations (i.e., co-activation rates) of power amplitude states (Fig. 1) in two sets of non-overlapping datasets from each patient with progressively longer durations. Our results demonstrate that the difference between within-frequency band correlations stabilize across all frequency bands after several hours of recordings in all subjects (Supplementary Fig. 1). Next, we examine the degree to which estimated correlations change as we incrementally increased the temporal span of each datasets. We hypothesize that the correlation values will become stable after several hours, after incorporating several hours of the available inter-ictal data from patients’ multi-day recordings. Figure 2 demonstrates that the changes in correlation matrices become progressively smaller and plateau on small values after several hours (≈12 h). We also note that the high *γ* band exhibits higher variability over time, indicating a requirement of longer recordings for stable estimation of correlations in 3 out of 5 patients (as seen in Fig. 2). Together, these results suggest that long iEEG recordings allow accurate estimation of the static functional connectivity between electrodes across a wide range of frequencies. Because multiple days of recordings are still theoretically insufficient to capture all 2^*N*^ possible states, this stabilization of co-activation rates indicates that the state space of practical co-activations is smaller than this theoretical limit. Therefore, we believe the pairwise MEM allows us to measure the strength of the essential relationships between sampled brain regions, which enables us to explain the frequencies (i.e., probabilities) of anticipated activation patterns.

### Pairwise MEM reveals high cross-frequency similarity in functional connectivity

**Fig. 3:**
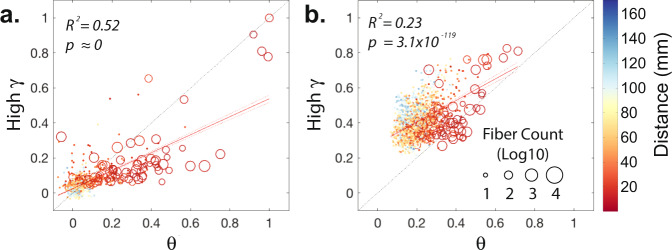
Cross-frequency similarity of the estimated functional interactions. **a** Normalized weights of pairwise MEM interaction matrices, *J*_*ij*_, estimated from the lowest (i.e., *θ*) and highest (i.e., high *γ*) frequency bands for a representative patient (#5). Edges from the interaction matrices with corresponding direct anatomical connections are marked by ‘o’ and their size indicates the number of estimated streamlines between each pair of regions. The functional edges without any detected anatomical connections are marked by ‘.’. Edges are color-coded based on the pairwise Euclidean distance between recorded brain regions. Red line represents the linear fit to the scatter plots (slope = 0.51 and intercept = 0.02 in panel **a**, slope = 0.61 and intercept = 0.28 in panel **b**) and each dashed red lines represent the 95% confidence interval. **b** Co-activation rates calculated from the same patient’s binarized band-passed power amplitude states at the lowest (i.e., *θ*) and highest (i.e., high *γ*) frequency bands.

Given our hypothesis that the inferred maximum entropy parameters should reflect the underlying structural connectivity, one would expect these pairwise interactions to maintain a similar architecture across a broad range of frequencies. Indeed, the matrix *J*_*ij*_, which encodes the inferred pairwise interactions between electrodes, displays notably high cross-frequency similarity. Although cross-frequency similarities are higher between the *J*_*ij*_ matrices estimated from more adjacent frequency bands, the average similarly values are high even between the lowest and the highest frequency bands (Supplementary Fig. 2). Scatter plots in Fig. 3a demonstrates the similarity between elements of the *J*_*ij*_ matrices estimated from a representative patient’s (#5) inter-ictal power amplitude states at *θ* and high *γ* frequency bands – See Supplementary Fig. 3 for all patients’ scatter plots. The fit of a simple linear regression model, which explains more than half of the variance, and the small intercept value of the model (slope = 0.51, intercept = 0.02, *p* ≈ 0 and *R*^2^ = 0.52) highlight the similarity between the interaction matrices.

As seen in Fig. 3b, the similarity between the co-activation rates at low- and high-frequency bands is relatively smaller, highlighted by the weak effect size of the linear regression model and its bigger intercept values (slope = 0.61, intercept = 0.28, *p* = 3.1 × 10^−119^, and *R*^2^ = 0.23). Note that the relatively high co-activation rates at high *γ* between distant brain regions without direct anatomical connectivity in Fig. 3b also limits using the co-activation rates at higher-frequency bands for predicting the structurally connected regions. The iEEG recordings are relative measures of electrical potentials against potential in another electrode and therefore the recording configuration (i.e., choice of reference electrode) or montage can effect the signal-to-noise levels (see Materials and Methods for details on recording configurations). We also find that although parameters such as the recording montage and state binarization threshold can notably impact the cross-frequency similarity of co-activation rates, the cross-frequency similarity of the estimated *J*_*ij*_ interaction matrices remain high across various parameters in all patients (Supplementary Fig. 2). Together, the pairwise MEM captures the intrinsic propensity for direct functional interactions between brain regions similarly across a wide range of frequencies.

### Pairwise MEM’s interaction weights reflect the white matter connectivity

**Fig. 4:**
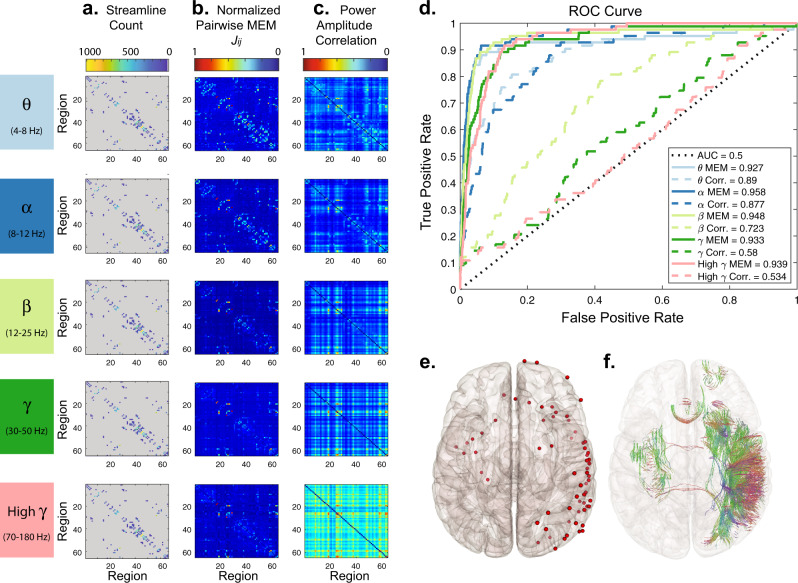
Predicting structural connectivity from estimates of functional connectivity. **a** A structural connectivity matrix from a single representative patient (#5), each element of which indicates the number of streamlines estimated between a pair of brain regions. **b** The interaction matrices, *J*_*ij*_, obtained from 19 h of inter-ictal iEEG power amplitude states (referential montage, binarization threshold = 0) using the pairwise MEM. **c** The functional connectivity matrices estimated from band-passed binarized power amplitude correlations (i.e., co-activation rates). For regions covered by multiple electrodes, we selected only the electrode closest to the region’s centroid for display in these plots as well as in the ROC analysis. **d** ROC curves for identification of anatomically connected regions based on interaction matrices (solid lines) and band-passed power amplitude correlations (dashed lines). Lines are color-coded based on the frequency band. **e** Brain overlay providing the position of the selected electrodes (red dots). **f** Streamlines connecting the regions covered by the selected electrodes in the same patient. Here the 3-dimensional orientations of the streamlines are coded by color.

Since the pairwise MEM takes the indirect propagation of influence between the electrodes into account, we hypothesized that compared to correlation, the distribution of estimated pairwise maximum entropy interaction weights to be heavy-tailed and skewed towards zero. Because of this explicit delineation of indirect and direct connectivity in the pairwise MEM, we hypothesized that it would improve functionally-derived estimates of direct structural connectivity. Consistent with our first hypothesis, we find that the *J*_*ij*_ weights are notably more heavy-tailed than the co-activation rates consistently across all frequency bands (Supplementary Fig. 4). The significantly (paired *t*-test, *p* < 0.05, *p* = 6.4 × 10^−7^, *N* = 5 patients × 5 frequency bands) high kurtosis of *J*_*ij*_ interaction weights’ distributions compared to that of the correlation values, also indicate the heavier tail of *J*_*ij*_ weight distributions (Supplementary Fig. 5). To test our second hypothesis, we examined the similarity between the functional and structural connectivity (i.e., log normalized streamline count) matrices, as well as the ability of the pairwise MEM interaction weights to predict underlying structural connectivity. Cross-modality correlations and prediction-based receiver operator characteristic (ROC) analyses both revealed a high degree of similarity between *J*_*ij*_ interaction matrices and structural connectivity. Figure 4 depicts ROC curves for identifying structural connectivity using *J*_*ij*_ interaction matrices and co-activation rates from the same representative patient in Fig. 3. The *J*_*ij*_ interaction matrices’ Area Under the ROC Curve (AUC) values in Fig. 4b and average cross-modality correlation of 0.56 ± 0.08 indicate the strong coupling between modalities across a wide range of frequency bands. We provide the AUC and cross-modality correlation values for all patients in Supplementary Figs. 6 and  7, respectively. We also note that overall across all patients, recording montages, and frequency bands, the cross-modality correlation on average is significantly higher (average difference in correlation coefficient *r* = 0.04, two-sided *t*-test, *p* = 1.03 × 10^−24^) after normalization (i.e, ${\mathrm{log}\,}_{10}$(streamline count + 1)) of streamline counts (Supplementary Fig. 8).

**Fig. 5:**
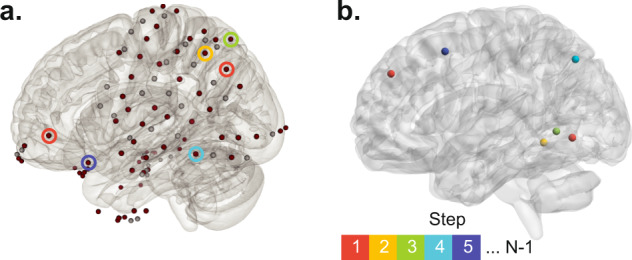
Schematic of geometric structural connectivity null algorithm. **a** The brain overlay shows the position of all electrodes from a sample patient (#5). Selected electrodes are colored-coded (dark red). **b** We create geometric null by resampling the patient’s brain regions. At the first step, we find two random distant brain regions and find a pair of empirically sampled brain regions such that the Euclidean distance between the empirical pair maximally matches that of the resampled null pair. The red circles in panel **a** highlight the empirically sampled electrodes and the center of corresponding null regions with red spheres. Next, out of all possible remaining 598 null regions of interest and the remaining *N* − 2 (*N* = total number of selected electrodes) empirically sampled regions, we find two brain regions, one from null and one empirical, with the most similar distances to the regions identified at the first step. At each following step, we repeat this process until we resample N null regions. Here we illustrate this procedure by showing an example of matched empirical (color-coded circles in panel **a**) and null (color-coded spheres in panel **b**) regions over five steps.

Extensive prior work has demonstrated that the weights of structural connections between brain regions decreases as a function of inter-regional distance^36–38^. The effects of distance on structural connectivity are potentially even more pronounced with respect to iEEG data because the electrode grids and strips are distributed focally rather than uniformly across the brain. Indeed, we observe that the most structurally connected electrode pairs are proximal and that long range fibers are scarce (Supplementary Fig. 9). In fact, the distance between electrodes is highly predictive of the presence of a structural connection between them (average AUC across subject = 0.986 ± 0.003). Finally, recording artifact such as volume conductance and presence of common sources disproportionately affect the estimated functional relationship between proximal regions. Therefore, to better account for distance-related artifacts, we adopted two strategies. First, we re-examined the results using two alternative recording montages to further account for global and local noise sources. Second, we compared all results against structural null models with equivalent distance profiles as the recoded brain regions. This enabled us to establish the robustness of our observations above and beyond distance-dependent relations. To create each structural null, we determined the structural connectivity between *N* (=number of empirically sampled brain regions) randomly resampled regions from the patient’s brain. To maintain the pairwise empirical distance profiles, we first select a random pair of distal null regions and identify the pair of empirical regions with the most similar pairwise distance. Next, the remaining null regions are identified iteratively such that at every iteration, the pairwise distances between the new null region and all null regions selected in previous iterations are maximally similar to those of its corresponding empirical region and all empirical regions identified at previous iterations (Fig. 5) – see the methods section Geometric structural connectivity null for more details.

**Fig. 6:**
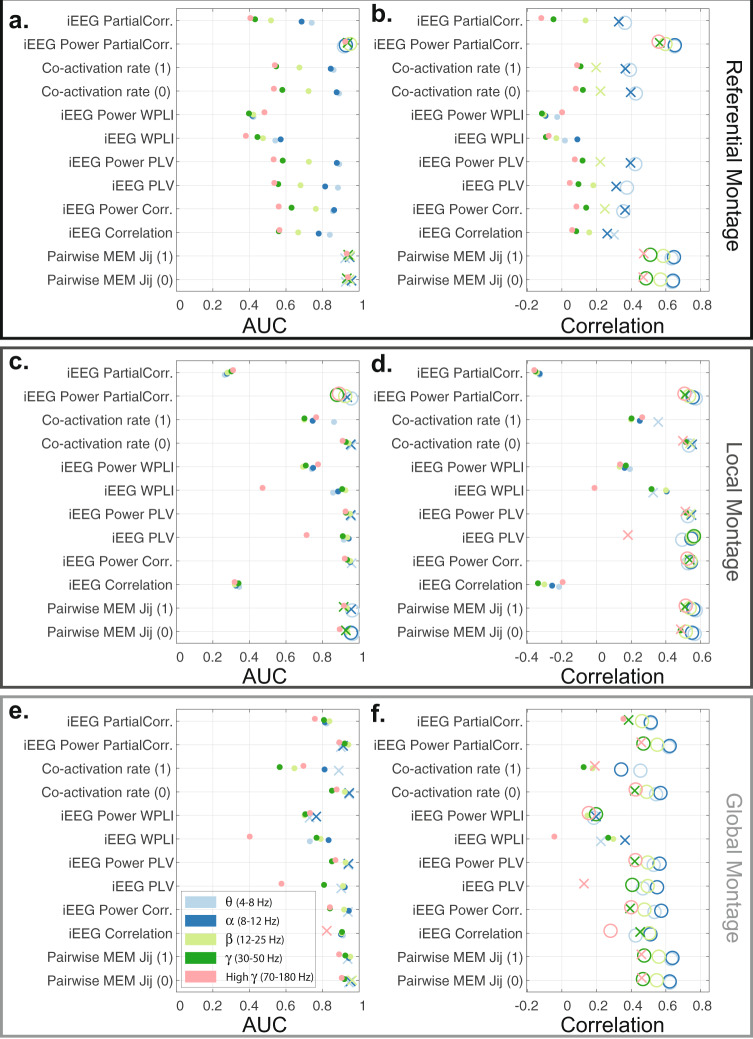
Similarity between structural (log normalized streamline count) and functional connectivity estimates. The detection accuracy (measure via AUC) of structural connectivity between brain regions using several functional connectivity estimation methods, or a representative patient (#5) using three recording montages -- referential **(a)**, local multiple linear regression **(c)**, and global mean signal regression **(e)** montages. **b, d, f** The structure-function coupling, measured as the correlation between structural and functional connectivity weights of sampled brain regions for the same patient and three recording montages. Functional connectivity estimates based on the pairwise MEM and co-activation rates were calculated from binarized (‘0’ and ‘1’) power amplitude states. The remaining functional connectivity estimates (Pearson correlation, partial correlation, PLV, and WPLI) were derived from the band-passed iEEG and band-passed iEEG power time series. The results for different frequency bands are color-coded based on the legend in panel **e**. The AUC and correlation values significantly higher than those of geometric structural nulls (one-sided permutation test, *n* = 10, 000, *p* < 0.05) are depicted as ‘X’, and values significant after FDR correction for multiple comparisons across frequency bands are depicted as ‘O’.

The similarity between the empirical structural and functional connectivity matrices estimated using the pairwise MEM is significantly higher than nulls in all frequency bands (one-sided permutation test, *n* = 10,000 *p* < 0.05, false discovery rate (FDR) corrected for multiple comparisons across all frequency bands), in at least one of the recording montages across all patients. We presented the difference between the empirical and null’s similarity (correlations) of the structural (log normalized streamline count) and functional connectivity matrices in Supplementary Fig. 10 using different recording montages. Also, we tested these results against a host of conventional functional connectivity methods including: Pearson correlation, partial correlation, phase-locking value (PLV), weighted phase-locking index (WPLI) using iEEG, and iEEG power time series (Supplementary Fig. 10). These methods provide converging results showing the high structure and function similarity using local or global montages, though the pairwise MEM and partial correlation of iEEG power reveal significant differences at a broader range of frequency bands and overall structure-function coupling. Similarly, we compared the ROC results for the detection of empirical and null structural connectivity matrices using different functional methods and recording montages in Supplementary Fig. 11. These results show that pairwise MEM and partial correlation of iEEG power provide the most accurate and significant (one-sided permutation test, *n* = 10, 000, *p* < 0.05, FDR corrected for multiple comparisons across all frequency bands) identification of anatomical connectivity from functional estimates in 3 out of 5 patients across several frequency bands. Our results also demonstrate that increasing the power amplitude states’ binarization threshold in general either yields comparable outcomes, or further enhances the structure-function coupling (as seen in Supplementary Figs. 10 and  11). We also note that unlike other methods, WPLI fails to reveal multi-modal agreement in regional coupling, which suggests the functional relevance of near zero-lag synchro’nizations between proximal brain regions. In Fig. 6 we provide the aforementioned results from a representative subject (#5). Together, these results suggest that, although the presence of structural connectivity is highly confounded with inter-regional distance, the pairwise MEM reveals high structure-function coupling across a broad range of frequencies, beyond those anticipated by inter-regional distance alone.

Next, to examine the relationship between the strength of structural and functional connectivity, we repeated the structure-function coupling analyses after removing a variable percentage of the weakest structural connections. We hypothesized that removing weak edges would increase the structure-function coupling in some patients, as false-positives in structural measurements are more likely to manifest as higher in weaker connections. Supplementary Fig. 12 shows the results from patient #1 after progressively removing a portion of weak structural edges. Although the outcomes vary to a degree across different recording montages, removing weak edges increased the structure-function coupling across all frequency bands in this patient. Supplementary Fig. 13 shows the result of a similar analysis for all patients using referential montage. These results also demonstrate that removing weak edges increased the correlation between structural and functional connectivity matrices in all patients (Supplementary Fig. 13b). At the same time, the ROC analyses in Supplementary Fig. 13a reveal our ability to detect structural connectivity does not improves after pruning weak structural edges, except in patient #1. Overall, these results demonstrate that more stringent structural connectivity estimations increases the measured structure-function coupling in some patients.

### Pairwise MEM accurately predicts co-activation states’ probabilities

We assessed the performance of the pairwise MEM first by comparing the empirically observed and activation and co-activation rates with those approximated by the model. As shown in Supplementary Fig. 14, the learning algorithm accurately reconstructs the activation and pairwise activation rates, well within experimental precision across all frequency bands and patients. These results demonstrate that the pairwise MEM provides a statistically robust prediction of the observed correlation structure of the power amplitude states across all frequency bands. Next we examined the goodness-of-fit of the pairwise MEM, which reveals how well a simple model using only a combination of the estimated activation rates and pairwise co-activation rates can predict the probability of the iEEG power amplitude states. One way to assess the goodness-of-fit fit for the pairwise MEM is to calculate the degree to which the estimated probabilities for the observed activity states diverge from the empirically measured probabilities. As a baseline, this divergence between the pairwise model and the observed statistics is often compared to the divergence of the simpler first-order model, which assumes that each brain region is behaving independently from the others. It is important to note that this calculation can be computationally expensive for large systems because the state space increases exponentially (2^*N*^) with the number of electrodes (*N*).

**Fig. 7:**
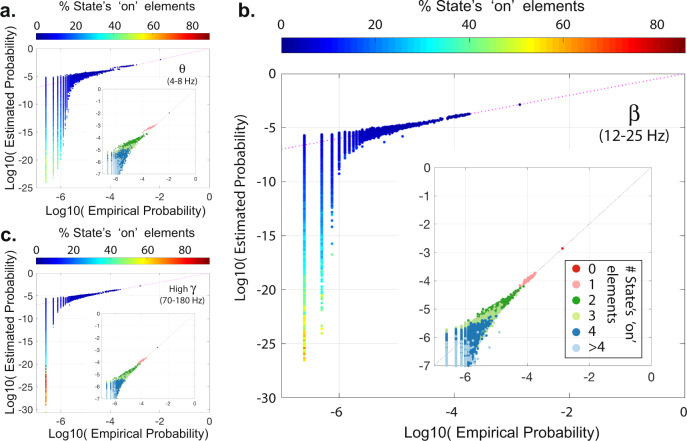
Empirical and estimated probabilities of all observed states. Plots represent the empirical and estimated probabilities of all observed states from a representative patient (#5) at *θ***(a)**, *β***(b)**, and High *γ***(c)** bands using local montage and state binarization threshold of ‘1’. The co-activation states’ size (i.e., the percentage ‘on’ elements of states) is color-coded based on the color bar on top of each panel. Insets depict the highest probability states within their respective frequency band. States in the inset-plots are also color-coded based on the number of ‘on’ elements of each state (see panel **b** for color-code legend). Supplementary Fig. 15 shows the results for all five patients.

To circumvent this issue, we compare the probability of all empirically observed states to their predicted probabilities. Calculating predicted’ probabilities usually is very difficult in high-dimensional datasets as calculating the normalization constant in the Boltzmann distribution (i.e., partition function) involves summing over all possible states. Therefore, we approximate the partition function using the empirical probability of the silent state (i.e., ‘all-off’) – see^33,39^ for more details. Scatter plots in Fig. 7 show the high similarity between the empirical and the estimated probabilities of all empirically observed states at *θ*, *β*, and high *γ* frequency bands from a representative patient (#5) using local recording montage binarized at 1. These results show that the pairwise MEM predicts the probabilities of the most frequently observed states with high accuracy. Similar results are also found in all five patients, as seen in Supplementary Fig. 15.

**Fig. 8:**
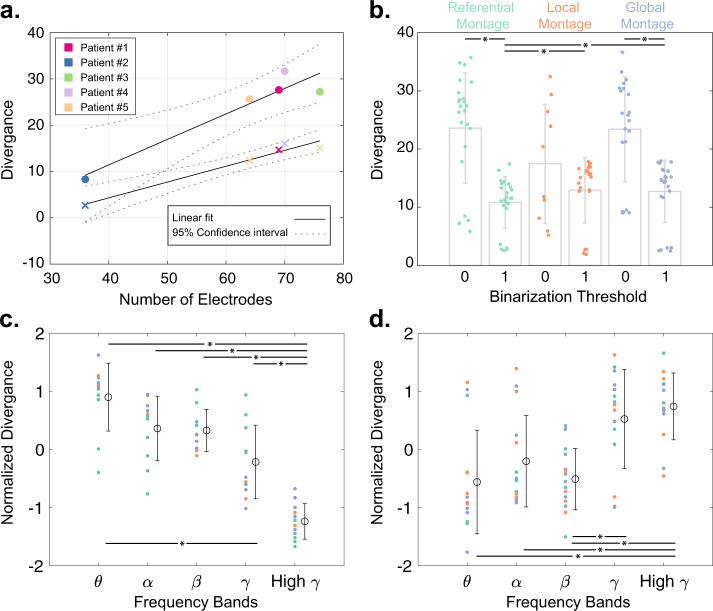
Goodness-of-fit of pairwise MEM. We measure the divergence between the empirically observed states’ empirical and estimated probabilities using a modified version of Kullback–Leibler divergence (see Materials and Methods section for details). **a** The relationship between the average (across all frequency bands, and recording montages) divergence of the empirical and estimated probabilities and the number of selected electrodes for each patient at ‘0’ (marked by ‘.’) and ‘1’ (marked by ‘x’) state binarization thresholds. The close fits of the linear regression models for both ‘0’ (slope = 0.55, *p* = 0.013, *R*^2^ = 0.9) and ‘1’ (slope = 0.34, *p* = 0.003, *R*^2^ = 0.96) thresholds suggest a negative relationship between the iEEG dimensions and the goodness-of-fit of pairwise MEM. **b** Bar plots show the average and standard deviation of divergence values for different recording montages and binarization thresholds (*n* = 5 patients × 5 frequency bands, except for the local montage states binarized at ‘0’ (*n* = 10) where the silent state (i.e., ‘all-off’) used for approximating the estimated probability function was observed only in 2 patients’ states). Statistical comparisons reveal that the divergence values are significantly smaller at higher binarization thresholds in the referential and the global montages (two-sided Wilcoxon rank-sum test, *p* < 0.05, marked by ‘*’). The divergence values are also lower in the referential montage compared to the other two montages only at the higher binarization threshold (two-sided Wilcoxon rank-sum test, *p* < 0.05, marked by ‘*’). **c, d** The normalized (i.e., z-scored across frequency bands) divergence values at different frequency bands for binarization thresholds of ‘0’ and ‘1’, respectively. Each dot represents a single patient, and their colors indicated the recording montage (color-coded similar to panel **b**). The open circles and error bars show the distributions’ mean and the standard deviation (*n* = 5 patients × 3 recording montages, with the above mentioned exception of local montage states binarized at ‘0’). Statistical comparisons reveal that the divergence values are significantly lower and higher at high *γ* compared to lower frequency bands using binarization thresholds of ‘0’ and ‘1’, respectively (two-sided Wilcoxon rank-sum test, *p* < 0.05, Bonferroni corrected for multiple comparisons, marked by ‘*’).

Our results also show that the goodness-of-fit of the pairwise MEM decreases linearly with the number of selected electrodes for each patient, as seen in Fig. 8a. We measure the goodness-of-fit by quantifying the divergence between the empirical and the estimated probabilities using a modified version of Kullback–Leibler divergence (see Materials and Methods section for details). We find a significant (two-sided Wilcoxon rank-sum test, *p* < 0.05) divergence of the predicted from empirical probabilities in iEEG power amplitude states binarized at 0 than 1 (Fig. 8b) using both referential and global mean regression montages. The goodness-of-fit is also significantly different between recording montages only at the higher (i.e., 1) binarization threshold. As seen in Fig. 8b, divergence values are significantly (two-sided Wilcoxon rank-sum test, *p* < 0.05) smaller in the referential montage than both local and global montages. Finally, we find significant differences in the pairwise MEM’s predication accuracy across low- and high-frequency bands. As seen in Fig. 8c and d, the pairwise MEM’s divergence from the empirical probabilities is significantly lower than low-frequency bands (two-sided Wilcoxon rank-sum test, *p* < 0.05, Bonferroni corrected for multiple comparisons) at binarization threshold of 0, and significantly higher than low-frequency bands at binarization threshold of 1. Together, these results demonstrate that the pairwise MEM’s accuracy is highest in predicting co-activation probabilities at low-frequency bands (i.e., *θ*, *α* and *β* bands).

## Discussion

A critical open question in neuroscience is how the relatively fixed white matter structure of the brain gives rise to complex functional dynamics. Some have argued that these complex dynamics are well-approximated by a single underlying process^40,41^, while others favor a model comprised of multiple processes that the brain can switch between^40,42,43^. In either scenario, it is critical to understand from a statistical perspective whether the observed dynamics can be explained by a fixed structural scaffold, such as that represented by the pattern of white matter fiber bundles connecting large-scale brain regions. The answer depends to some degree on whether or not one can identify statistical features of the functional dynamics that display relatively time-invariant properties. Some evidence for such time-invariance has recently been obtained from eletrocorticography, where patterns of functional connectivity between electrodes appear relatively stable in windows larger than a few minutes^17,18,44^. Our observations also provide converging evidence that the correlation patterns and, consequently, the intrinsic pairwise relationships between brain regions that give rise to power amplitude dynamics, are effectively stable in long multi-hour recordings. Thus, these findings further motivate a careful investigation into the correspondence between those functional relationships and the underlying anatomical projections.

A second line of evidence supporting the existence of a common structural scaffold for the observed dynamics is the presence of notable similarity in the estimated pairwise interactions across frequency bands. This statistical similarity is reminiscent of the cross-frequency interactions that have been observed in other studies, and that are thought to play a role in integrating information across brain structures that are functionally specialized and spatially segregated^45–47^. It is commonly thought that the diverse rhythms present in different frequency bands are associated with different spatio-temporal scales of neural activity^48,49^, with low frequencies driving activity over larger spatial areas and high frequencies driving activity over smaller spatial areas^50^. Our work offers a useful complement to these prior studies by demonstrating that the pairwise MEM provides higher cross-frequency similarity in estimated interactions than conventional measures of functional connectivity. Future work could examine the real-time dynamics of cross-frequency coupling by combining power amplitude states across all bands in a single model. Taken together, our observations of high cross-frequency similarity indicate that a common underlying physiological^51^ or anatomical^52^ mechanism is likely driving the functional interactions between brain regions across all frequency bands.

We validate the possibility that patterns of functional interactions track an underlying structural scaffold, by showing the similarity between structural connectivity and pairwise MEM interaction matrices. We demonstrate that strength of interactions can be used to predict the underlying white matter structure in the same patients across a wide range of frequency bands, consistent with prior work in other imaging modalities^29^. It is of interest to determine the contribution of distance to structure-function coupling. The strength of a structural connection between two brain regions tends to be negatively correlated with the distance between them, as does the strength of the functional connection between them^35^. One possibility is that common sources that are simultaneously measured by nearby electrodes drive the structure-function coupling. Therefore, we established the significance of our findings by rigorous statistical comparisons against structural null models, which we created by resampling patients’ brain regions with distance profiles similar to the recorded brain regions. Inline with our prior findings^53^, we also show that alternative functional connectivity methods such as PLV^54^ provide converging results at several frequency bands. Nevertheless, compared to phase- and correlation-based methods, the pairwise MEM and partial correlation of iEEG power time series produced the biggest divergence from the null and revealed significant structure-function coupling even at higher frequency bands. Inline with to prior work by Watanabe et al.^29^, these results provide evidence that accounting for global patterns of pairwise interactions likely reduces the spurious correlations and contributes to the partial correlation and pairwise MEM’s ability to uncover the multi-modal coupling. Together, our observations suggests that the functional dynamics captured by the pairwise MEM extends beyond common sources or local spreading phenomena, and that the presence of a structural connection between brain regions likely plays a crucial role in shaping the emergent functional activity across a wide range of frequencies.

Our results also demonstrate that overall the functional connectivity estimated using pairwise MEM and partial correlation of iEEG power time series across all three montages reveals significant coupling to the underlying white matter connectivity between the recorded regions across a wide range of frequencies. Other functional connectivity methods such as PLV and pairwise correlations provide convergent results, only after spatial filtering, usually from *θ* to *γ* (4–50 Hz) frequency bands. These observations highlight the relative insensitivity of the pairwise MEM to shared reference noise. In general, however, our results show that local montage results in higher similarity and more robust detection of structural connections across more patients and at higher frequencies, which suggests that volume conductance effects are major source of artifacts that negatively impact structure-function coupling estimations. Our results also show the poor performance of the WPLI measure, which highlights the functional relevance of near zero-lag synchronizations.

Pairwise MEMs have been employed effectively to explain patterns of collective neural activity across a range of spatial scales^26,29,31^. A good fit of the pairwise MEM indicates that the observed patterns of power amplitude states can be accurately described with a combination of first-order activations and second-order (pairwise) co-activation between electrodes. A poor fit signifies that higher-order effects (e.g., triplet or quadruplet interactions between regions) or that common inputs from other external regions may be required to explain the behavior of the system. Here, we demonstrate that the pairwise MEM allows us to accurately approximate the empirical correlation patterns of power amplitude states in several biologically relevant frequency bands. Moreover, we find that the pairwise MEM can predict the frequency (i.e., probability) of the empirically observed co-activation patterns with relatively high accuracy in all patients. Our results also suggest that the difference in sampling likely contributes to the observed inter-subject variability, as the accuracy of the model correlates negatively with the number of examined brain regions in each patient. Overall, we demonstrate that the goodness-of-fit of the model – the degree of divergence between the observed and predicted probabilities – is highest at the higher iEEG power amplitude binarization threshold (i.e., one) and low-frequency bands (i.e., *θ*, *α*, *β* bands). These results suggest that higher thresholds improve the fit of the model likely by increasing the signal-to-noise ratio. We expect some approximation error, as the likelihood of empirically observing the lower probability states is incredibly low. In fact, we observe the biggest probability estimation errors in the least frequently observed states. In addition, the relatively longer inter-ictal dataset needed for a stable estimation of iEEG power functional connectivity (i.e., co-activation rates), likely contributes to the larger errors at high *γ* band.

Nevertheless, we speculate that the relatively higher error at low binarization threshold (i.e., zero) and higher frequency bands might also originate from biophysical mechanisms contributing to nearby electrodes’ co-activation. Examples of such biophysical mechanisms include the spatially distributed activation sources and wave patterns, including traveling and spiral waves^55,56^. It is important to note that even though iEEG electrodes cover a large portion of the cortical surface in these patients, we are still only able to capture a small fraction of the full system, and it is highly likely that some amount of co-activation is driven by input from external sources that are not covered by an electrode. Although the MEM could be extended to include higher-order parameters beyond the pairwise interactions considered here (e.g.,^39^), the practical limitation of partial brain coverage and the previously noted biophysical mechanisms combine to hinder the interpretability of higher-order mechanisms. Together, our findings suggest that a simple pairwise MEM allows us to explain how the strength of the intrinsic interactions between brain regions gives rise to the observed functional connectivity structure and shape the frequency of emerging co-activation patterns.

One common concern regarding the use of iEEG datasets from patients with epilepsy to study the functional organization of the human brain is their documented aberrations of structural connectivity as well as structure-function coupling^15,57,58^. One could argue that aspects of our results dependent on such aberrations may not generalize, as they may not reflect the structure-function relationship in healthy brains. Since iEEG datasets from healthy controls do not exist, we cannot directly address these concerns. Nevertheless, we draw on recent work demonstrating that the patterns of iEEG functional connectivity in patients show statistical similarities to structural connectivity estimated in healthy volunteers^52^, and that this statistical similarity is upheld and even strengthened during ictal epochs^59,60^. Future work using data from patients with other pathologies, or using source-localized MEG in healthy patients, could be helpful in further understanding the nature of the structure-function correspondence accessible to the MEM.

Our results reveal that the functional connectivity estimated using the pairwise MEM corresponds closely to the underlying structural connectivity between sampled regions, and we tested the significance of these observations using conservative structural null models. Although preprocessing methods and parameter choices can impact estimates of structural connectivity, we observed that the correspondence between functional interactions and structural connections were robust to reasonable variation in these choices. Future work could test the robustness of our findings to other important preprocessing parameters such as region size and contemporary streamline filtration techniques.

We show that our proposed geometric structural null preserves the distribution of the distances between regions, with a relatively small degree of error. We further demonstrate that given our current experimental design, this amount of error is within the tolerance range, as the average distance between iEEG electrodes and the centroid of their most proximate ROIs were significantly larger. Future work can aim to reduce the former by biasing the geometric null algorithm towards regions with a high distance error (e.g., by initializing the algorithm with a small set of empirical ROIs with high distance errors), and the latter type of error by defining electrode-centric ROIs. It is worth noting that our results show that the distance error is larger only in a few electrodes, which suggests that the idiosyncratic electrode placements likely contribute to the systematic divergence of the null distance profiles.

Our observations have potentially important implications for understanding large-scale functional brain dynamics as well as our ability to modulate these dynamics via stimulation or resection. Our findings are consistent with the notion that the pairwise MEM may be particularly sensitive to structurally-driven functional relations while conventional functional connectivity methods may be sensitive to non-structurally-driven functional relations that might vary appreciably over short time intervals. The observed high degree of structure-function coupling suggests that structural connectivity is a useful proxy for time-invariant functional relationships^52,59^. This observation could be useful in the treatment of epilepsy patients, where access to the brain is traditionally limited to recording loci but could be augmented with non-invasive measurements of structural connectivity for more informed surgical planning^60^. Indeed, it is intuitively plausible that computational models built to inform the modulation of abnormal functional dynamics via stimulation^61^ or resection^62^ may be able to utilize patient-specific structural connectivity in place of or to augment patient-specific functional connectivity.

## Materials and methods

### Patient information

Five patients (mean age 41.6, standard deviation 4.8; 3 female) undergoing surgical treatment for medically refractory epilepsy at the Hospital of the University of Pennsylvania underwent implantation of subdural electrodes for localization of the seizure onset zone. All patients had unilateral temporal lobe epilepsy, determined by comprehensive clinical evaluation and validated by seizure free one-year outcomes following temporal lobectomy. This study was approved by the Institutional Review Board of the University of Pennsylvania, and all subjects provided written informed consent prior to participating.

### Intracranial EEG acquisition

De-identified patient data was downloaded from the online International Epilepsy Electrophysiology Portal (iEEG Portal, http://www.ieeg.org)^63^. iEEG signals were recorded at 512 Hz at the Hospital of the University of Pennsylvania, Philadelphia, PA. Subdural electrode (Ad Tech Medical Instruments, Racine, WI) configurations consisted of linear strip and two-dimensional grid arrays (2.3 mm diameter with 10 mm inter-contact spacing). Inter-ictal periods were selected to be at least 6 h away from clinically marked seizures.

### Recording configurations

iEEG recordings are relative measures as they measure the electrical potential against potential in another electrode. Usually, this reference electrode is placed far from the recording site on the brain or bone. This recording configuration is referred to as referential montage, and in the past, has been utilized in two-dimensional local field potential recordings^64,65^. However, since the reference electrode itself contains signal fluctuations, it can contaminate the activations at the recording electrode. This common noise is either ignored (e.g.,^66^) or extracted by re-referencing to a global signal average (e.g.,^53,67^). This global montage is referred to as the common average reference.

However, if the profile of the noise is less homogeneous, for instance, due to the considerable distance between electrodes in stereotactic EEG (sEEG) recordings, references such as local Laplacian montage can be used for noise reduction via local spatial filtering^68,69^. Laplacian montages also suppress the volume conductance effects by removing the potentials accounted for by the local references^34^. Nevertheless, due to this property, local Laplacian montage can also introduce error, as they strongly attenuate the broadly-distributed co-activations patterns.

In this study we used a referential montage utilizing a reference electrode distant to the seizure onset zone for recording iEEG signals. Also, we explored two alternative recording configurations. First, to account for common reference noise, we implemented a global average reference montage by regressing out the global mean signal from individual electrodes’ time series. Second, in the same vein as Laplacian montages^68^, we regressed out the effect of local noise (such as volume conductance) by fitting a multiple linear regression model to each electrode’s iEEG time series using the time series of four closest electrodes as explanatory variables. Despite the fundamental similarity between local multiple linear regression and local weighted averaging montages, local multiple linear regression does not explicitly account for distance differences of each electrode to the other four selected local electrodes. Nevertheless, this property of local multiple linear regression allows us, in theory, to remove the locally inhomogeneous noise (i.e., noise shared between only a few local electrodes) more effectively.

### Image acquisition

MRI data were acquired using a 3-Tesla Siemens scanner (Siemens Magnetom Trio Tim Syngo MR B17, Germany) at the Hospital of the University of Pennsylvania. Diffusion-weighted images were acquired in a single-shot echo-planar imaging, multi-shell protocol (2.5 × 2.5 × 2.5 mm^3^ resolution, TR = 5216 ms, TE = 100 ms, FOV = 220 mm, MB acceleration factor = 2, flip/refocus angle = 90/180°, phase encoding direction = anterior to posterior). A total of 119 volumes were acquired at *b* = 0 (16 volumes), *b* = 300 (8 volumes), *b* = 700 (31 volumes), and *b* = 2000 (64 volumes). T1-weighted MPRAGE images were also obtained for each subject (0.94 × 0.94 × 1.0 mm^3^ resolution, TR = 1810 ms, TE = 3.51 ms, FOV = 240 mm, flip angle = 9°, phase encoding direction = right to left).

### Structural connectivity

T1-weighted MPRAGE images were used to co-register MNI-space atlases to subject structural space via AntsRegistrationSyN^70^. Similarly, we used the first b0 image from each patient’s diffusion sequence to calculate co-registration transforms from subject-specific diffusion to structural space. Using this set of transforms, we brought individualized AAL 600 atlases^7^ for all five patients into diffusion space for tractography. Atlas to diffusion co-registrations were performed using ANTs and FSLs FLIRT. Eddy current and motion corrections were performed using FSLs 5.0.9 EDDY patch. To mitigate susceptibility distortions, we used the subjects MPRAGE T1 structural scan. This image was first brought to diffusion space by using the inverse of our FLIRT transformation for each subject, and then contrast-inverted and intensity-matched to the DWI image using FSLMATHS. Finally, the DWI image was non-linearly transformed to the shape of the MPRAGE scan, as these acquisitions are not subject to the same susceptibility distortions observed in DWIs.

We evaluated several distinct methodological approaches, and we also assessed the consistency of our findings across them. We performed deterministic and probabilistic tractography in Camino^71^ on distortion-corrected DWIs to construct adjacency matrices for structural connectivity. Deterministic tractography parameters were stringent so we could err on the side of caution in evaluating the physiological feasibility of our reconstructed white matter connections. These parameters included removing estimated fibers in areas of the brain where 90% or greater of diffusion was estimated to be isotropic versus restricted^72^, where fractional anisotropy was determined to be below 0.05, or where a T1-derived brain mask ended. We also removed fibers that curved more than 50° over 5 mm to again restrict fiber reconstructions to physiological feasibility. Finally, we removed fibers shorter than 10 mm to minimize spurious short-range connections based on only a few voxels, and we also removed fibers over 400 mm to prevent looping artifactual fibers from impacting our results. Structural adjacency matrices were constructed from the number of streamlines that begin and end in each pre-defined ROI. We employed equivalent parameters for probabilistic tractography and utilized the PICO algorithm^73^. Processing power limited us to seeding 400 times per voxel. Within deterministic and probabilistic tractography, we evaluated streamline count (SC) weighted adjacency matrices.

### Pairwise maximum entropy model

To form unbiased predictions for the probabilities of various functional brain states, we fit a pairwise maximum entropy model, which is motivated by the principle of maximum entropy. The principle of maximum entropy states that when estimating a probability distribution given some desired constraints, one ought to consider the distribution that maximizes the uncertainty (i.e., entropy); choosing any other distribution that lowers the entropy would assume additional information beyond our desired constraints. Fitting the pairwise MEM entails tuning the first and second-order interaction parameters between regions so that the predicted activation rates and co-activation rates match the empirically observed values. An accurate fit of the pairwise MEM implies that the observed patterns of collective activity can be understood as emerging from each region’s independent activation rate combined with regions’ joint activation rates. In other words, the pairwise MEM allows us to establish a model of iEEG functional power dynamics as a probabilistic process shaped by underlying pairwise relationships between brain regions.

Our use of the power amplitude envelope to define the activation states is motivated by several factors. First, it has been shown that the BOLD fMRI signal echoes the envelope of high-frequency neural activity^74,75^, specifically when measured by iEEG^52,59,76,77^. Thus, in light of studies linking BOLD fMRI functional connectivity to white matter structural connectivity^6,29^, we hypothesize that the power of iEEG recordings should also exhibit a clear relationship with the underlying structural connectivity. Second, a large body of evidence has demonstrated that iEEG oscillations play an important role in healthy cognitive function, and that breakdowns in oscillatory power are linked to cognitive disorders^78,79^. Third and finally, by defining our activity states using band-passed power amplitudes, we gain the ability to analyze patterns of activity across both time and frequency, thereby mitigating inconsistent phase relationships between electrodes caused by spatially non-stationary signal sources, such as iEEG spiral waves^55^. However, recent modeling work suggests that the metastable wave-like patterns are also able to recapitulate the empirically observed functional connectivity patterns, if they are plentiful on longer time scales^56^.

Following prior works, such as^80^, we define ‘on’-‘off’ activation states for each brain region by binarizing the normalized envelope of the power amplitude of each frequency band (Fig. 1). We fit the pairwise MEM to the thresholded and binarized normalized power amplitude states of electrodes from concatenated 14, 16, 14, 13, and 19 h of inter-ictal iEEG recording of patients 1–5, respectively. We picked only the single closest (Euclidean distance) electrode to the ROI’s center for analysis when more than one electrodes exist within the same ROI. A total of 69, 36, 76, 70, and 64 electrodes were selected from patients 1–5, respectively. To calculate the power amplitude states, we first segmented the time-series into 36 100-second windows (Fig. 1a). Next, for each segment and each electrode we calculated the wavelet power^81^ of the time-series (Fig. 1b). We band-passed the wavelet power by averaging the power amplitude across all the frequencies within each band. Next, the power amplitude time-series (all bands) were high-pass filtered at 0.5 Hz to minimize the effect of ultra-slow fluctuations of power on the binarized states. The high-passed time series were then *z*-scored and binarized by thresholding at zero to create band-passed power amplitude states (Fig. 1c). We also explored higher binarization threshold of one. Finally to reduce the temporal dependence of samples we down-sampled the states by the factor of 10. At each time *t*, the power amplitude state is defined by ${V}^{t}=\left[{\sigma }_{1}^{t},{\sigma }_{2}^{t},...,{\sigma }_{N}^{t}\right]$, where ${\sigma }_{i}^{t}$ is the binarized power amplitude of electrode *i* at time *t*, where ${\sigma }_{i}^{t}=1$ (0) for power above (below) the threshold, and *N* is the total number of electrodes. For electrode *i*, the empirical power amplitude activation rate $\left\langle {\sigma }_{i}\right\rangle$ is given by $(\frac{1}{T})\mathop{\sum }\nolimits_{t = 1}^{T}{\sigma }_{i}^{t}$, where *T* is the number of time slices. Likewise, the empirical co-activation rate between electrodes *i* and *j*, $\langle {\sigma }_{i}{\sigma }_{j}\rangle$, is defined by $(\frac{1}{T})\mathop{\sum }\nolimits_{t = 1}^{T}{\sigma }_{i}^{t}{\sigma }_{j}^{t}$.

Here, our only constraints were that the model averages ${\left\langle {\sigma }_{i}\right\rangle }_{m}$ and ${\langle {\sigma }_{i}{\sigma }_{j}\rangle }_{m}$ matched the empirical values of $\left\langle {\sigma }_{i}\right\rangle$ and $\langle {\sigma }_{i}{\sigma }_{j}\rangle$, respectively. Given these constraints, it is known that the probability distribution that maximizes the entropy is the Boltzmann distribution^25^:

1 $$P({V}_{k})={e}^{-E({V}_{k})}/\mathop{\sum }\limits_{q=1}^{{2}^{N}}{e}^{-E({V}_{q})},$$

where *P*(*V*_*k*_) is the probability distribution of the *k*th state *V*_*k*_, and *E*(*V*_*k*_) is the energy of this state, which is given by:

2 $$E({V}_{k})=-\mathop{\sum }\limits_{i=1}^{N}{h}_{i}{\sigma }_{i}({V}_{k})-\frac{1}{2}\mathop{\sum }\limits_{i,j=1}^{N}{J}_{ij}{\sigma }_{i}({V}_{k}){\sigma }_{j}({V}_{k}),$$

where *σ*_*i*_(*V*_*k*_) is the value of *σ*_*i*_ for state *V*_*k*_, *h*_*i*_ represents the expected base power amplitude rate of electrode *i* in isolation, and *J*_*ij*_ represents the functional interaction between electrodes *i* and *j*.

Fitting the pairwise MEM entails iterative adjustment of the parameters *h*_*i*_ and *J*_*ij*_ with a gradient descent algorithm^30^ until the empirical averages $\left\langle {\sigma }_{i}\right\rangle$ and $\langle {\sigma }_{i}{\sigma }_{j}\rangle$ match those in the model, namely ${\left\langle {\sigma }_{i}\right\rangle }_{m}=\mathop{\sum }\nolimits_{q = 1}^{{2}^{n}}{\sigma }_{i}({V}_{q})P({V}_{q})$ and ${\langle {\sigma }_{i}{\sigma }_{j}\rangle }_{m}=\mathop{\sum }\nolimits_{q = 1}^{{2}^{n}}{\sigma }_{i}({V}_{q}){\sigma }_{j}({V}_{q})P({V}_{q})$. Since the state space is prohibitively large in our data, we followed^82^ by approximating the model averages ${\left\langle {\sigma }_{i}\right\rangle }_{m}$ and ${\langle {\sigma }_{i}{\sigma }_{j}\rangle }_{m}$ by calculating the correlation of a sequence of random samples (*N* = 10,300,000, after discarding the first 300,000 and downsampling by a factor of 500) from the probability distribution using the Metropolis–Hastings algorithm^83^. However, our preliminary analysis reveals that the error in the approximated activation rates is notably high. Therefore, instead of the aforementioned likelihood maximization approach, alternatively, we utilized the pseudo-likelihood maximization algorithm using MATLAB scripts provided by Ezaki et al.^32^ to estimate the parameters of the model. In pseudo-likelihood maximization scheme, we aim to solve

3 $$(h,J)=\arg \mathop{\max }\limits_{h,j}{\mathcal{L}}(h,J),$$

where the pseudo-likelihood function, ${\mathcal{L}}(h,J)$, is defined as

4 $${\mathcal{L}}(h,J)\approx \mathop{\prod }\limits_{t=1}^{{t}_{max}}\mathop{\prod }\limits_{i=1}^{N}\tilde{P}({\sigma }_{i}| h,J,{\sigma }_{/i}(t))\left.\right),$$

Here, $\tilde{P}$ represents the Boltzmann distribution for a single spin (i.e., electrode), *σ*_*i*_, given that the other *σ*_*j*_(*j* ≠ *i*) values are fixed to *σ*_/*i*_(*t*) ≡ (*σ*_1_(*t*), ..., *σ*_*i*−1_(*t*), *σ*_*i*+1_(*t*), ..., *σ*_*N*_(*t*)). Therefore, $\tilde{P}$ is given by

5 $$\tilde{P}({\sigma }_{i})=\frac{{e}^{({h}_{i}{\sigma }_{i}+\mathop{\sum }\nolimits_{i,j = 1}^{N}{J}_{ij}{\sigma }_{i}{\sigma }_{j}(t))\left.\right)}}{{\sum }_{{\sigma }_{i}^{\prime} = 1,0}{e}^{({h}_{i}{\sigma }_{i}^{\prime}+\mathop{\sum }\nolimits_{i,j = 1}^{N}{J}_{ij}{\sigma }_{i}^{\prime}{\sigma }_{j}(t))\left.\right)}},$$

This mean-field approximation neglects the influence of *σ*_*i*_ on *σ*_*i*_ (*j* ≠ *i*), however, the estimator afforded by the maximization of the pseudo-likelihood converges to the maximum-likelihood estimator as *t*_*max*_ → *∞*^84^. The parameters of the model, *h* and *J*, are estimated using a gradient ascent scheme given by

6 $${h}_{i}^{new}-{h}_{i}^{old}=\epsilon ({\left\langle {\sigma }_{i}\right\rangle }_{empirical}-{\left\langle {\sigma }_{i}\right\rangle }_{\tilde{P}})$$

and

7 $${J}_{ij}^{new}-{J}_{ij}^{old}=\epsilon ({\langle {\sigma }_{i}{\sigma }_{j}\rangle }_{empirical}-{\langle {\sigma }_{i}{\sigma }_{j}\rangle }_{\tilde{P}}),$$

Where the superscripts new and old represent the parameters after and before a single updating step, respectively, *ϵ* (>0) is the learning rate, and ${\left\langle {\sigma }_{i}\right\rangle }_{\tilde{P}}$ and ${\langle {\sigma }_{i}{\sigma }_{j}\rangle }_{\tilde{P}}$ are the mean and correlation with respect to distribution $\tilde{P}$ (Eq. (5)) and are given by

8 $${\left\langle {\sigma }_{i}\right\rangle }_{\tilde{P}}=\frac{1}{{t}_{max}}\mathop{\sum }\limits_{t = 1}^{{t}_{max}}\tanh \left[{h}_{i}+\mathop{\sum }\limits_{j^{\prime} = 1}^{N}{J}_{ij^{\prime} }{\sigma }_{j^{\prime} }(t)\right]$$

and

9 $${\langle {\sigma }_{i}{\sigma }_{j}\rangle }_{\tilde{P}}=\frac{1}{{t}_{max}}\mathop{\sum }\limits_{t=1}^{{t}_{max}}{\sigma }_{j}(t)\tanh \left[{h}_{i}+\mathop{\sum }\limits_{j^{\prime} =1}^{N}{J}_{ij^{\prime} }{\sigma }_{j^{\prime} }(t)\right],$$

respectively. For more details regarding the pseudo-likelihood maximization scheme see^32^. As seen in Supplementary Fig. 14, the approximated activation and co-activation rates match the empirical values within the experimental precision. It is worth noting that the reconstruction of activation and co-activation rates using the gradient ascent method is trivial in the maximum-likelihood method since the problem is concave with respect to the *J* and *h* parameters^32^. However, for the pseudo-likelihood maximization method used here, convergence to the correct solution where the activation and pairwise co-activation rates in the model match those in the empirical data (Supplementary Fig. 14).

The co-activation states can also be defined as $\tilde{{\sigma }_{i}}\in \left\{-1,1\right\},(i=1,...,N)$ instead of ${\sigma }_{i}\in \left\{0,1\right\}$, since mathematically, the energy function representations are equivalent and have a one-to-one relationship; $2{\sigma }_{i}-1=\tilde{{\sigma }_{i}}$. Therefore the pairwise MEM parameters, *J*_*ij*_ and *h*_*i*_, also have the following relationships:

10 $${h}_{i}=2\tilde{{h}_{i}}-2\mathop{\sum }\limits_{i=1}^{N}\tilde{{J}_{ij}}$$

11 $${J}_{ij}=4\tilde{{J}_{ij}}$$

Since we used the method from^32^ to estimate the model parameters that define the states as $\tilde{{\sigma }_{i}}\in \left\{-1,1\right\}$, we transform the estimated $\tilde{{J}_{ij}}$ and $\tilde{{h}_{i}}$ matrices following Eqs. (10) and (11), respectively. Usually, calculating the normalization constant in the Boltzmann distribution (i.e., *partition function*) in high-dimensional datasets is very difficult, as it involves summation over all possible states. This transformation allows us to approximate the partition function ($\hat{Z}$) directly from the probability of the *silent* state (i.e., *p*(000⋯0)) as $\hat{Z}=\frac{1}{p(000\cdots 0)}$, since it holds that $p(000\cdots 0)=\frac{1}{Z}$^33,39^.

### Empirical and estimated probabilities divergence measure

To examine the goodness-of-fit of the pairwise MEM, in the same vein as^39^, we measure the divergence between the empirically observed states’ empirical and estimated probabilities using a modified version of Kullback–Leibler measure defined as:

12 $$D(P,Q)=\sum _{x}P(x)\left|{\mathrm{log}\,}_{2}\left(\frac{P(x)}{Q(x)}\right)\right|$$

where *P*(*x*) and *Q*(*x*) are the empirical and estimated probabilities of state *x*. Unlike the original Kullback–Leibler measure, we use the absolute value of the log. Since the estimated probabilities are not normalized, it is important to penalize states where the model overestimates their probabilities, as they will reduce the Kullback–Leibler divergence values.

### Phase-base functional connectivity methods

Originating from the field of physics, studying synchronizations in the brain has been instrumental in expanding our understanding of its functional organization. In this framework, two brain regions are considered synchronized if their electrophysiological recordings exhibit stable phase differences across time or trials. Introduced first by Lachaux et al.^54^, the phase-locking value (PLV) measure calculates the consistency in the difference between the instantaneous phase of two band-passed signals. Formally, PLV is defined as:

13 $$PLV=\left|E\left\{{e}^{i\theta }\right\}\right|$$

where $E\left\{.\right\}$ is the expectation operator, and *θ* the difference between the instantaneous phase values of two filtered iEEG times series. We determine the instantaneous phase using a Hilbert transform from the band-passed time series. PLV values range from zero to one, signifying no to full synchrony (i.e., consistent phase-difference), respectively. In theory, volume conductance, the signal from a source simultaneously recorded by two nearby electrodes, would result in apparent synchronization with a phase difference of 0 (or *π* depending on the position of the dipole). Phase-locking index (PLI) was introduced^85^, which aims to reduce sensitivity to volume conductance by disregarding phase-locking with 0 or *π* phase difference. PLI is formally defined as:

14 $$PLI=\left|E\left\{sgn\left(\Im \left\{X\right\}\right)\right\}\right|$$

where $sgn\left(\Im \left\{X\right\}\right)$ is the sign of the imaginary part of the cross-spectrum of two signals *S*_1_ and *S*_2_ defined as:

15 $$X\equiv {Z}_{1}{Z}_{2}^{* }$$

where *Z*_1_ and *Z*_2_ are vectors of complex-valued Fourier spectra, obtained using the Discrete Fourier Transform of *S*_1_ and *S*_2_ time series, and ${Z}_{2}^{* }$ is the complex conjugate of *Z*_2_. A weighted version of this measure was later introduced by Vinck et al.^86^, based on downweighting the influences of couplings with phase differences close to 0 or *π*, on the final phase-locking values. The Weighted Phase-locking Index (WPLI) is defined as:

16 $$WPLI=\frac{\left|E\left\{\Im \left\{X\right\}\right\}\right|}{E\left\{\left|\Im \left\{X\right\}\right|\right\}}$$

Note that as long as two signals maintain a positive (or negative) phase difference at all time points, WPLI will return one indicating highest synchrony. Thus, in theory, WPLI reduces false positives as it will not overestimate connectivity due to volume conduction. However, WPLI can miss true connections due to small lags or frequency non-stationarities^87^.

### Partial correlation

Partial correlation coefficient (*ρ*) between any two variables *X*_*i*_ and *X*_*j*_ of a set **V**, given all others variables (i.e., **V**⧹{*X*_*i*_, *X*_*j*_}) can be calculated from the inverse covariance matrix (*p*) as:

17 $${\rho }_{{X}_{i}{X}_{j}\cdot {\bf{V}}\setminus \{{X}_{i},{X}_{j}\}}=-\frac{{p}_{ij}}{\sqrt{{p}_{ii}{p}_{jj}}}$$

However, we used MATLAB’s partialcorr.m scripts, which instead computes partial correlation coefficient, ${\rho }_{{X}_{i}{X}_{j}}$, as the correlation of the residuals from the regression of *X*_*i*_ and *X*_*j*_ on **V**⧹{*X*_*i*_, *X*_*j*_}. We calculated the partial correlation matrices from hour-long iEEG and iEEG power time series segments. Similar to the pairwise MEM analysis, we downsampled the EEG and iEEG power time series prior to calculating partial correlations by a factor of 10 to reduce the temporal dependence of samples. Finally, for each patient, we averaged the partial correlation matrices calculated from all hour-long segments

### Geometric structural connectivity null

iEEG electrodes are about one centimeter from their neighboring electrodes. This high density of iEEG electrodes allows us to cover a patch of cortex with a large number of electrodes. However, this high density also increases the possibility of artificially inflating functional connectivity between proximal electrodes due to factors such as common source signals or volume conductance issues. Therefore, to tease out the intrinsic functional relationship for the mentioned recording artifacts expected from nearby electrodes, prior work has proposed several methods to account for the distance between the measured brain regions. For instance, to remove the volume conductance effects and radially symmetric correlations, some groups have suggested adjusting the functional connectivity using distance regression strategy^88^. More recently, other geometric structural nulls have been introduced that aim to maintain the higher-order statistical structure of the edge weights based on the distance profile^89^.

Here, we introduce a new paradigm based on iterative resampling of individual patients’ brain regions aimed at maximizing the similarity between null brain region distances and recorded brain region distances. This process is akin to virtual resampling of the patient’s brain with the same number of electrodes with similar geometry, as it maintains the distance profile of each pair of electrodes. This method also benefits from its minimal assumptions on the expected relationship between structural connectivity weight and distance. Instead, it takes advantage of each patient’s whole-brain structural connectome and the patient-specific geometry of iEEG electrodes’ placement, to create individualized structural connectivity nulls with conserved distance profiles.

Specifically, we first calculate the distance between all pairs of brain regions. We initiate the algorithm by randomly sampling a pair of brain regions, with distances close to the most distant pair of the recorded brain regions. Next, we identify the two brain regions, one null and one recorded region, which are located the most similarly relative to the pair of null and recorded brain regions identified at the first step, respectively. This process is repeated, on brain regions at a time, until all the recorded brain regions and their corresponding nulls are matched.

It is worth noting that the idiosyncrasies of patients’ electrode placements yield variable regional distance profiles, which should be considered in the initiation step of the algorithm. For instance, since most edges between the recorded brain regions are short to mid-range, the algorithm produces comparable distributions when initiated with the less frequent distance brain regions. In Supplementary Fig. 16, we provide details regarding each patient’s structural connectivity null and their distance profiles. These results demonstrate that the estimated null models closely match the distance profiles of the recorded brain regions, though some null brain regions are located with a small degree of error relative to other brain regions. Nevertheless, as seen in Supplementary Fig. 17, we demonstrate that this error is within the tolerance range, as it is smaller than error associated with the distance between iEEG electrodes, and the centroid of their most proximate ROIs. As seen in Supplementary Fig. 18, statistical testing reveals that the empirical distance between electrodes and the center of their corresponding ROI is significantly larger than the average null regions’ distance errors (i.e., the mismatches between null and recorded brain regions) across all patients (two-sided *t*-test, *p* < 0.05, *p* = 9.73 × 10^−12^, *p* = 3.16 × 10^−13^, *p* = 9.65 × 10^−14^, *p* = 2.6 × 10^−16^, *p* = 3.29 × 10^−13^). In Fig. 5, we provide the schematic representation of our proposed resampling algorithm for generating geometric structural connectivity nulls. We also provide the pseudocode of our proposed resampling algorithm in the Supplementary Methods.

### Predicting anatomical connectivity from functional connectivity

We conducted ROC analysis to determine the accuracy with which the strength of the functional interactions in the pairwise MEM predict the presence of structural connectivity. We constructed the ROC curves to represent the true positive (structurally connected brain regions with functional interaction weight above the threshold) and false positive (structurally disconnected brain regions with functional interaction weight above the threshold) at different detection thresholds. The area under the curve (AUC) of the ROC plot represents the accuracy of the classification, where an AUC value of 1 indicates perfect classification, and an AUC value of 0.5 indicates classification performance at the chance level.

### Statistics and reproducibility

We used non-parametric permutation tests to compare the coupling between the recorded brain regions’ anatomical and functional connectivity against null models that maintain the distance profile between electrode pairs. We tested the relationship between the recording montage, number of iEEG electrodes, and frequency bands with the pairwise MEM’s goodness-of-fit (measured as the divergence between empirical and predicted co-activation probability) using the Wilcoxon rank-sum test. We used *t*-test to compare the empirical distance between the electrode and the ROI center to the distance error of nulls across all patients. We also used paired *t*-test for statistical comparisons between the kurtosis values calculated from *J*_*ij*_ interaction weight distributions and those of correlation distributions. We used Bonferroni and false discovery rate (FDR) to correct for multiple comparisons throughout the manuscript.

### Reporting summary

Further information on research design is available in the Nature Research Reporting Summary linked to this article.

## Supplementary information

Description of Additional Supplementary Files

Supplementary Data 1

Supplementary Data 2

Reporting Summary

Supplementary Information

## Data Availability

All inter-ictal iEEG data are available from the International Epilepsy Electrophysiology Portal (IEEG-Portal, http://www.ieeg.org). Information about datasets used in this manuscript are listed in Supplementary Data 1.

## Code availability

The custom scripts for generating geometric structural null models are available at https://github.com/aashourv/iEEG_MEM.
