# Supplementary material for: Pairwise maximum entropy model explains the role of white matter structure in shaping emergent co-activation states
Source: Commun Biol. 2021 Feb 16;4:210. doi: 10.1038/s42003-021-01700-6 (PMC7887247; doi:10.1038/s42003-021-01700-6)
Supplement: Supplementary file 1 — Description of Additional Supplementary Files [file 42003_2021_1700_MOESM1_ESM.pdf]

Description of Additional Supplementary Files

File name: Supplementary Data 1

Description: Information about datasets from the IEEG-Portal, http://www.ieeg.org

File name: Supplementary Data 2

Description: Source Data for Figures

Manu_Fig3_Panel_a_Data

Manu_Fig3_Panel_b_Data

Manu_Fig4_Panel_a_SC_Data

Manu_Fig4_Panel_b_alpha_Data

Manu_Fig4_Panel_b_beta_Data

Manu_Fig4_Panel_b_gamma_Data

Manu_Fig4_Panel_b_Hgamma_Data

Manu_Fig4_Panel_b_theta_Data

Manu_Fig4_Panel_c_alpha_Data

Manu_Fig4_Panel_c_beta_Data

Manu_Fig4_Panel_c_gamma_Data

Manu_Fig4_Panel_c_Hgamma_Data

Manu_Fig4_Panel_c_theta_Data

Manu_Fig6_Panel_a_c_e_Data

Manu_Fig6_Panel_b_d_f_Data

Manu_Fig7_Panel_a_Data

Manu_Fig7_Panel_b_Data

Manu_Fig7_Panel_c_Data

Manu_Fig8_Panel_a_Data

Manu_Fig8_Panel_b_Data

Manu_Fig8_Panel_c_Data

Manu_Fig8_Panel_d_Data
