# Supplementary material for: Pairwise maximum entropy model explains the role of white matter structure in shaping emergent co-activation states
Source: Commun Biol. 2021 Feb 16;4:210. doi: 10.1038/s42003-021-01700-6 (PMC7887247; doi:10.1038/s42003-021-01700-6)
Supplement: Supplementary file 4 — Reporting Summary [file 42003_2021_1700_MOESM4_ESM.pdf]

nature research

Corresponding author(s): BDPR COMMSBIO-19-0999C

Last updated by author(s): Dec 11, 2020

Reporting Summary

Nature Research wishes to improve the reproducibility of the work that we publish. This form provides structure for consistency and transparency in reporting. For further information on Nature Research policies, see our Editorial Policies and the Editorial Policy Checklist.

Statistics

For all statistical analyses, confirm that the following items are present in the figure legend, table legend, main text, or Methods section.

n/a	Confirmed	
☐	☒	The exact sample size (*n*) for each experimental group/condition, given as a discrete number and unit of measurement
☐	☒	A statement on whether measurements were taken from distinct samples or whether the same sample was measured repeatedly
☐	☒	The statistical test(s) used AND whether they are one- or two-sided<br>*Only common tests should be described solely by name; describe more complex techniques in the Methods section.*
☐	☒	A description of all covariates tested
☐	☒	A description of any assumptions or corrections, such as tests of normality and adjustment for multiple comparisons
☐	☒	A full description of the statistical parameters including central tendency (e.g. means) or other basic estimates (e.g. regression coefficient) AND variation (e.g. standard deviation) or associated estimates of uncertainty (e.g. confidence intervals)
☐	☒	For null hypothesis testing, the test statistic (e.g. *F*, *t*, *r*) with confidence intervals, effect sizes, degrees of freedom and *P* value noted<br>*Give P values as exact values whenever suitable.*
☒	☐	For Bayesian analysis, information on the choice of priors and Markov chain Monte Carlo settings
☒	☐	For hierarchical and complex designs, identification of the appropriate level for tests and full reporting of outcomes
☐	☒	Estimates of effect sizes (e.g. Cohen's *d*, Pearson's *r*), indicating how they were calculated

Our web collection on statistics for biologists contains articles on many of the points above.

Software and code

Policy information about availability of computer code

Data collection	The data examined in this paper was primarily collected for clinical purposes. The clinical recording software for Penn Medicine is Natus Neuroworks XLTEK. Data is collected from the clinical servers using Natus platform. The data was then converted from Natus proprietary file format to mef2 format using a converter, which is protected under non-disclosure agreement between CNT and Natus. The converted filed were then uploaded to iEEG-portal (http://www.iEEG.org). For more details regarding the iEEG Portal see 10.1109/NER.2013.6696201
Data analysis	For data analysis, we used MATLAB 2019a and 2014b. We provide the costume codes for calculating the geometric structural connectivity null models in https://github.com/aashourv/iEEG_MEM.

For manuscripts utilizing custom algorithms or software that are central to the research but not yet described in published literature, software must be made available to editors and reviewers. We strongly encourage code deposition in a community repository (e.g. GitHub). See the Nature Research guidelines for submitting code & software for further information.

Data

Policy information about availability of data

All manuscripts must include a data availability statement. This statement should provide the following information, where applicable:

- Accession codes, unique identifiers, or web links for publicly available datasets
- A list of figures that have associated raw data
- A description of any restrictions on data availability

All inter-ictal iEEG data are available from the International Epilepsy Electrophysiology Portal (IEEG-Portal, http://www.ieeg.org). Information about datasets used in this paper are listed in Supplementary Table 1.

Field-specific reporting

Please select the one below that is the best fit for your research. If you are not sure, read the appropriate sections before making your selection.

☒ Life sciences ☐ Behavioural & social sciences ☐ Ecological, evolutionary & environmental sciences

For a reference copy of the document with all sections, see nature.com/documents/nr-reporting-summary-flat.pdf

Life sciences study design

All studies must disclose on these points even when the disclosure is negative.

Sample size	We analyzed an average of more than 14 hours (per patient) of inter-ictal iEEG recording of five patients. We demonstrate that the pairwise correlation between iEEG electrodes stabilize after several hours (>12 hours) of recording, which suggest that the length of dataset is sufficient for capturing the fundamental structure essential to the collective behavior in the recorded regions using pairwise MEM.
Data exclusions	We downloaded 24 one-hour segments of inter-ictal iEEG recordings from the online International Epilepsy Electrophysiology Portal (iEEG portal, http://www.ieeg.org) for each patient and after visual inspection removed all one-hour segments with artifacts.
Replication	We were able to replicate the pairwise MEM's goodness-of-fit results across all five patients. Similarly we found significant correlation between the pairwise MEM's interaction weights and the direct anatomical connectivity between recorded brain regions.
Randomization	We did not group the subjects in anyway. We provided subject-specific analysis and null models to verify our findings. Therefore, randomization was not relevant.
Blinding	Since we are not grouping the subjects as mentioned above, blinding was not relevant.

Reporting for specific materials, systems and methods

We require information from authors about some types of materials, experimental systems and methods used in many studies. Here, indicate whether each material, system or method listed is relevant to your study. If you are not sure if a list item applies to your research, read the appropriate section before selecting a response.

Materials & experimental systems

n/a	Involved in the study	
☒	☐	Antibodies
☒	☐	Eukaryotic cell lines
☒	☐	Palaeontology and archaeology
☒	☐	Animals and other organisms
☐	☒	Human research participants
☒	☐	Clinical data
☒	☐	Dual use research of concern

Methods

n/a	Involved in the study	
☒	☐	ChIP-seq
☒	☐	Flow cytometry
☐	☒	MRI-based neuroimaging

Human research participants

Policy information about studies involving human research participants

Population characteristics	Five patients (mean age 41.6, standard deviation 4.8; 3 female) undergoing surgical treatment for medically refractory epilepsy at the Hospital of the University of Pennsylvania underwent implantation of subdural electrodes for localization of the seizure onset zone. All patients had unilateral temporal lobe epilepsy, determined by comprehensive clinical evaluation and validated by seizure free one-year outcomes following temporal lobectomy.
Recruitment	Participants are recruited from patients undergoing surgical treatment. All patients provided written informed consent prior to participating.
Ethics oversight	This study was approved by the Institutional Review Board of the University of Pennsylvania.

Note that full information on the approval of the study protocol must also be provided in the manuscript.

Magnetic resonance imaging

Experimental design

Design type	We examined resting inter-ictal iEEG recording from all patients.

Design specifications	In this work we examined the coupling between the structural and functional connectivity estimated for each patient. Therefore there are no trials or conditions in this experimental framework
Behavioral performance measures	Patients did not perform any task and therefore there are no behavioral performance measurements in this study.

Acquisition

Imaging type(s)	Structural, Diffusion
Field strength	3 Tesla
Sequence & imaging parameters	MRI data were acquired using a 3-Tesla Siemens scanner (Siemens Magnetom Trio Tim Syngo MR B17, Germany) at the Hospital of the University of Pennsylvania. T1-weighted MPRAGE images were obtained for each subject (0.94x0.94x1.0 mm3 resolution, TR = 1810 ms, TE = 3.51 ms, FOV = 240 mm, flip angle = 9 degrees, phase encoding direction = right to left).
Area of acquisition	Whole brain

Diffusion MRI ☒ Used ☐ Not used

Parameters	Diffusion-weighted images were acquired in a single-shot echo-planar imaging, multi-shell protocol (2.5x2.5x2.5 mm3 resolution, TR = 5216 ms, TE = 100 ms, FOV = 220 mm, MB acceleration factor = 2, flip/refocus angle = 90/180 degrees, phase encoding direction = anterior to posterior). A total of 119 volumes were acquired at b=0 (16 volumes), b=300 (8 volumes), b=700 (31 volumes), and b=2000 (64 volumes).

Preprocessing

Preprocessing software	FSL, Ants, camino.
Normalization	T1-weighted MPRAGE images were used to co-register MNI-space atlases to subject structural space via AntsRegistrationSyN (https://doi.org/10.1016/j.media.2007.06.004). Similarly, we used the first b0 image from each patient's diffusion sequence to calculate co-registration transforms from subject-specific diffusion to structural space. Using this set of transforms, we brought individualized AAL 600 atlases (https://doi.org/10.1073/pnas.1219562110) for all five patients into diffusion space for tractography. Atlas to diffusion co-registrations were performed using ANTs and FSLs FLIRT.
Normalization template	MNI152
Noise and artifact removal	Eddy current and motion corrections were performed using FSLs 5.0.9 EDDY patch. To mitigate susceptibility distortions, we used the subjects MPRAGE T1 structural scan. This image was first brought to diffusion space by using the inverse of our FLIRT transformation for each subject, and then contrast-inverted and intensity-matched to the DWI image using FSLMATHS. Finally, the DWI image was non-linearly transformed to the shape of the MPRAGE scan, as these acquisitions are not subject to the same susceptibility distortions observed in DWIs.
Volume censoring	There was no volume censoring of the diffusion images

Statistical modeling & inference

Model type and settings	t-tests, Wilcoxon rank-sum test, non-parametric permutation tests for comparing distributions of null models to observed data, linear regression.
Effect(s) tested	We compare the coupling between the anatomical and functional connectivity of the recorded brain regions against null models that maintain the distance profile between electrode pairs. We also tested the relationship between the recording montage, number of iEEG electrodes, and frequency bands with the pairwise MEM's goodness-of-fit (measured as the divergence between empirical and predicted co-activation probability).

Specify type of analysis: ☐ Whole brain ☒ ROI-based ☐ Both

Anatomical location(s)	We used Automated Anatomical Labeling ('AAL') Atlas 600 (https://doi.org/10.1073/pnas.1219562110) to define our anatomical regions of interest.
Statistic type for inference (See Eklund et al. 2016)	Functional analyses were performed at iEEG electrode space.
Correction	We used FDR to correct for multiple comparisons across all possible pairs of electrodes.

Models & analysis

n/a	Involved in the study
☐	☒ Functional and/or effective connectivity
☒	☐ Graph analysis
☒	☐ Multivariate modeling or predictive analysis

Functional and/or effective connectivity	Pairwise MEM, Pearson correlation, Partial correlation, Phase-locking value (PLV), Weighted phase-locking index (WPLI).
