# Supplementary material for: Pairwise maximum entropy model explains the role of white matter structure in shaping emergent co-activation states
Source: Commun Biol. 2021 Feb 16;4:210. doi: 10.1038/s42003-021-01700-6 (PMC7887247; doi:10.1038/s42003-021-01700-6)
Supplement: Supplementary file 5 — Supplementary Information [file 42003_2021_1700_MOESM5_ESM.pdf]

Supplementary Information

Pairwise maximum entropy model explains the role of white matter structure in shaping emergent co-activation states

Arian Ashourvan[1,2,†,*], Preya Shah[1,2], Adam Pines[1], Shi Gu[3], Christopher W. Lynn[4], Danielle S. Bassett[1,4,5,6,7], Kathryn A. Davis[1,2], and Brian Litt[1,2,7]

[1]Department of Bioengineering, School of Engineering and Applied Science, University of Pennsylvania
[2]Penn Center for Neuroengineering and Therapeutics, University of Pennsylvania
[3]Department of Computer Science and Engineering, University of Electronic Science and Technology of China
[4]Department of Physics & Astronomy, School of Arts & Sciences, University of Pennsylvania
[5]Department of Electrical and Systems Engineering, School of Engineering and Applied Science, University of Pennsylvania
[6]Department of Psychiatry, Perelman School of Medicine, University of Pennsylvania
[7]Department of Neurology, Hospital of the University of Pennsylvania
[†]Correspondence to: Arian Ashourvan, 210 S. 33rd Street, Philadelphia, PA 19104-6321,
Email: ashourv@seas.upenn.edu

Contents

Supplementary Methods 3
Supplementary Figures 5

Supplementary Methods

Geometric structural connectivity null resampling algorithm pseudocode

Algorithm 1 Geometric structural connectivity null resampling algorithm

$N(=10000)$ ▷ Number of resampled structural nulls
$n_{Atlas}(=600)$ ▷ Number of anatomical Regions of Interests (ROIs), Automated Anatomical Labeling (AAL600) atlas
$D_{Atlas}(n_{Atlas} \times n_{Atlas})$ ▷ Matrix of Euclidean distances between anatomical ROIs' centroids
$iEEG\_ROIs$ ▷ list of unique ROIs closets to the center of iEEG electrodes
n_{iEEG_ROIs} ▷ Number of $iEEG\_ROIs$
$D_{iEEG}(n_{iEEG\_ROIs} \times n_{iEEG\_ROIs})$ ▷ Matrix of Euclidean distances between selected iEEG electrodes
$Repeat\_Ratio$=0.1; ▷ Threshold ratio of the same null ROI selected for an electrode across iterations
$Flag_{Repeat} = 0$ ▷ Initialize partial repeated sampling flag
$Flag_{Repeat}\_2 = 0$ ▷ Initialize full repeated sampling flag
procedure ITERATIVE RESAMPLING OF ROIS(D_{Atlas}, D_{iEEG},N)
    $Err_{Low} = 10$ (mm) ▷ Low distance error limit
    $Err_{High} = 10$ (mm) ▷ High distance error limit
    for Iteration =1 to N **do**
        $Flag_{initial} = 0$
        while $Flag_{initial} = 0$ **do** ▷ Ensure the distance between selected ROI pair is close to the most distance iEEG pair
            Null_ROIs(1,Iteration)= i_1 ▷ Select a random pair of ROIs, i_1 and i_2 from anatomical atlas
            Null_ROIs(2,Iteration)= i_2
            if $\max(D_{iEEG}) - Err_{Low} <$ Euclidean distance between i_1 and i_2 $< \max(D_{iEEG}) - Err_{High}$ **then**
                $Flag_{initial} = 1$
                Find the pair of iEEG electrodes j_1 and j_2 with a pairwise distance closest to that of ROIs i_1 and i_2
                Null_iEEG(1,Iteration)= j_1
                Null_iEEG(2,Iteration)= j_2
            end if
            if i_1 and $i_2 \in iEEG\_ROIs$ || Either ROIs are found more frequently than $Repeat\_Ratio$ across Iterations **then**
                $Flag_{initial} = 0$
            end if
        end while
        if Null_ROIs(:,Iteration) ∈ Null_ROIs(:,1:Iteration-1) **then**
            $Flag_{Repeat} = 1$
        end if
        for k = 1 to $n_{iEEG\_ROIs} - 2$ **do**
                ▷ From the remaining ROIs and electrodes find one ROI and one electrode with closest distance profiles to the selected null ROIs and their corresponding electrodes, respectively. Repeat until all electrodes are assigned a null ROI label.
            for roi =1 to n_{Atlas}) **do**
                for $electrode$ =1 to n_{iEEG_ROIs} **do**
                    if $roi \notin$ Null_ROIs & $electrode \notin iEEG\_ROIs$ **then**
                        $D_{Dissimilarity}(roi\ ,\ electrode)$ = Distance (Euclidean) between the vector of distances between roi to Null_iEEG to that of distances between $electrode$ to $iEEG\_ROIs$
                    end if
                end for
            end for

Algorithm 2 Geometric structural connectivity null resampling algorithm (Continued)

Find $\text{argmin}_{i_k, j_k} D_{Dissimilarity}$
Null_ROIs(2+k,Iteration)= i_k
Null_iEEG(2+k,Iteration)= j_k
if $Flag_{Repeat} = 1$ **then** ▷ If Null_ROIs are selected in previous Iterations choose the second best match
$D_{Dissimilarity}(i_k, j_k)$= Nan
Find $\text{argmin}_{i_k, j_k} D_{Dissimilarity}$
Null_ROIs(2+k,Iteration)= i_k
Null_iEEG(2+k,Iteration)= j_k
if $Flag_{Repeat}\_2 = 1$ **then** ▷ If previous Iterations include all second best matches choose the third best match
$D_{Dissimilarity}(i_k, j_k)$= Nan
Find $\text{argmin}_{i_k, j_k} D_{Dissimilarity}$
Null_ROIs(2+k,Iteration)= i_k
Null_iEEG(2+k,Iteration)= j_k
end if
end if
if Either ROIs are found more frequently than $Repeat\_Ratio$ across iterations **then** ▷ Find the next best match
$D_{Dissimilarity}(i_k, j_k)$= Nan
Find $\text{argmin}_{i_k, j_k} D_{Dissimilarity}$
Null_ROIs(2+k,Iteration)= i_k
Null_iEEG(2+k,Iteration)= j_k
end if
if Null_ROIs(:,Iteration) ∈ Null_ROIs(:,1:Iteration-1) **then**
$Flag_{Repeat} = 1$
else
$Flag_{Repeat} = 0$
end if
end for
▷ Flag if the entire Null_ROIs(:,Iteration) vector is found in previous iterations
if Null_ROIs(:,Iteration) ∈ Null_ROIs(:,1:Iteration-1) **then**
$Flag_{Repeat}\_2 = 1$
end if
end for
end procedure
▷ Null_ROIs and Null_iEEG outputs contain resampled null ROIs and their corresponding iEEG electrodes, respectively

Supplementary Figures

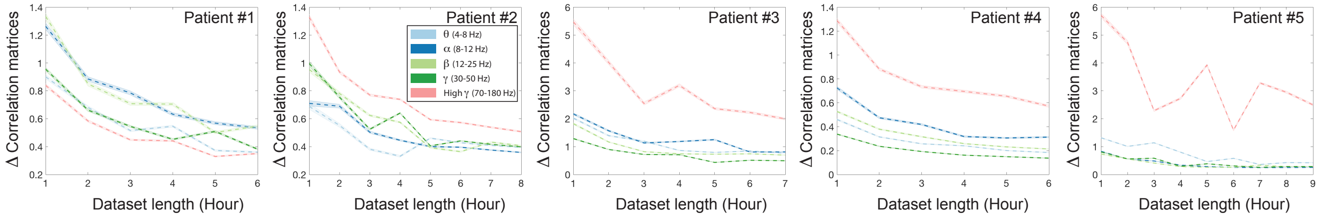

Figure 1. The relationship between length of dataset and stability of pairwise correlations. Plots show the mean (n =10,000 iterations) difference between the correlation (i.e., co-activation rates) matrices, calculated from the power amplitude states of two same length iEEG datasets, consisting of non-overlapping randomly selected one-hour inter-ictal segments. We use the matrix norm to quantify the difference between correlations matrices. The shaded-area around the means (dashed line) highlights the standard error, and colors indicate the frequency bands.

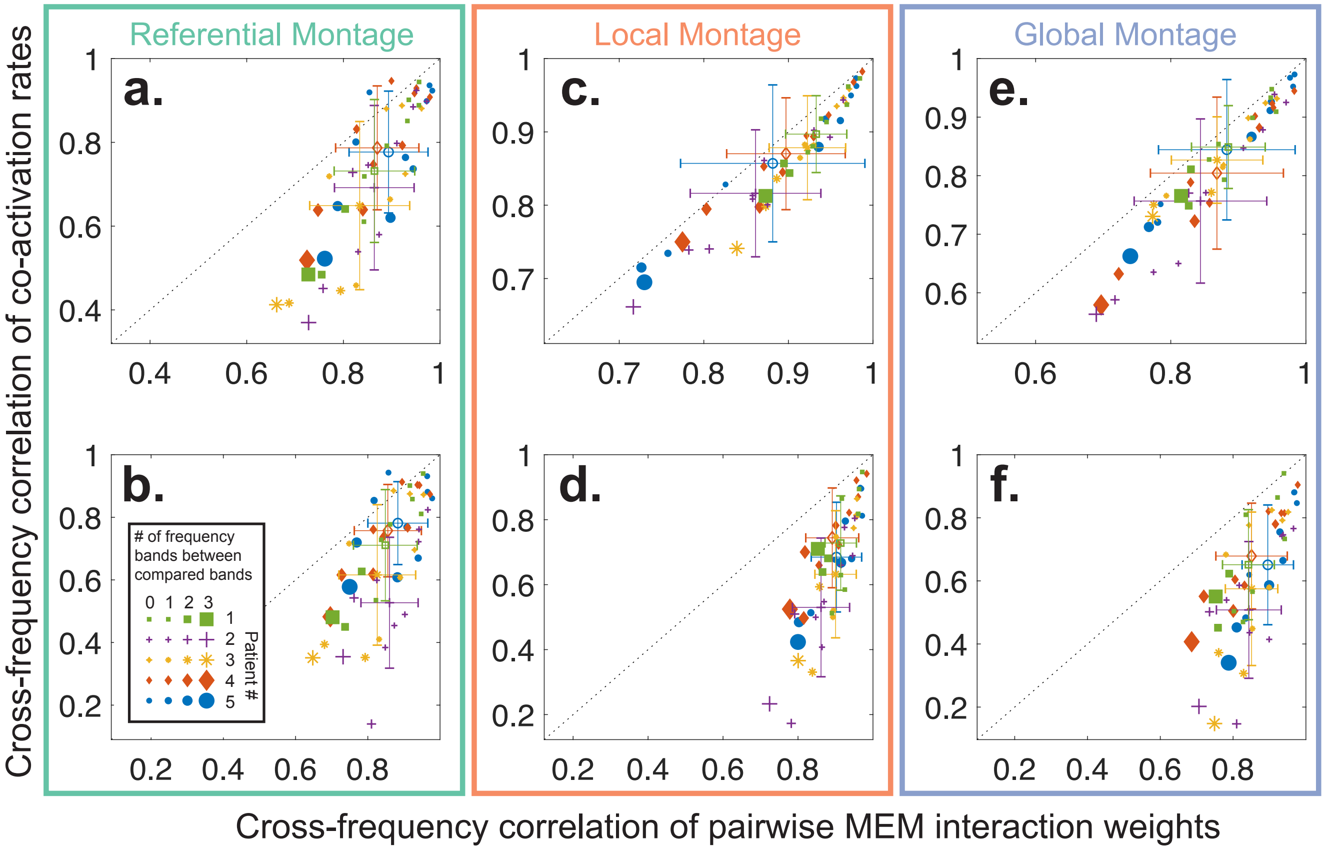

Figure 2. Similarity of pairwise maximum entropy interactions across frequencies. Each point in the scatter plot represents the cross-frequency similarity (i.e., Pearson correlation) of the J_{ij} interaction matrices (x-axis) against the cross-frequency similarity of the co-activation rates (y-axis), calculated from inter-ictal data across all possible pairs of frequency bands. The size shows how many other frequency bands are between the compared pair. The smallest markers indicate the comparisons between adjacent pairs of frequency bands (e.g., α and β), and the largest markers indicate comparison between the most distant pair of frequency bands, θ and high γ. Color-coded error-bars represent the mean and standard deviation of the correlation values across all possible pairs of frequency bands. The data from each patient is color-coded (see legend in panel ***b***). The dashed line represents the unity line. The plots on the top (panels ***a***, ***c***, and ***e***) and bottom (panels ***b***, ***d***, and ***f***) rows show the results for the power amplitude binarization threshold of 0 and 1, respectively. The plots on each column represent the results of referential, global mean regression, and local multiple regression montages, from left to right. Note that the hourly averages calculated from the J_{ij} matrices are relatively high (with mean >0.8) in all plots. We also note that although the co-activation rates also display high cross-frequency similarity when the power amplitude time series are thresholded at zero after re-referencing (panels ***c*** and ***e***). However, the cross-frequency similarity of co-activation rates reduces substantially at a higher binarization threshold of 1, as indicated by the significantly lower average cross-frequency values compared to that of pairwise MEM across all patients (two-sided Wilcoxon rank-sum test, $p < 0.05$) in panels ***d*** and ***f***. Note that the systematic shift from diagonals highlight that the cross-frequency similarity decreases much more rapidly between co-activation rates than that of the J_{ij} matrices for the distant frequency bands. Despite similar trends in referential montage results, pairwise MEM interaction matrices show significantly higher average cross-frequency similarity only in patients #3 and #4 in panels ***a*** and ***b***.

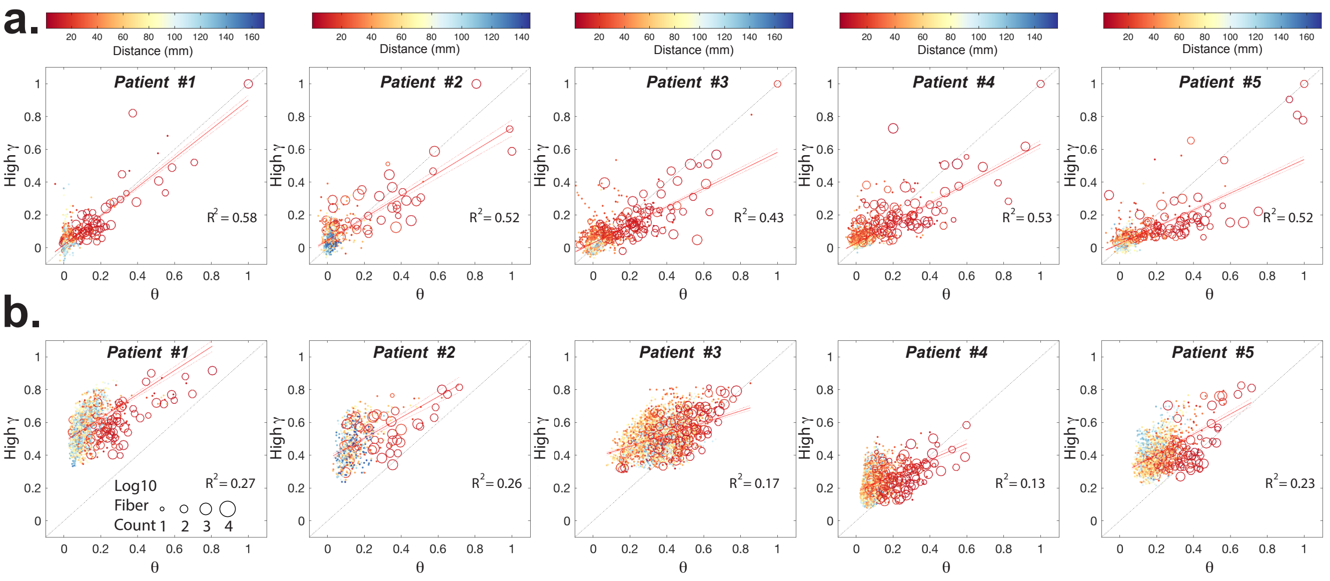

Figure 3. **Cross-frequency similarity of the estimated functional interactions** ***(a)*** Each point in the scatter plots represents the normalized weight of an edge from the pairwise MEM interaction matrices, J_{ij}, estimated from the lowest (i.e., θ) and highest (i.e., high γ) frequency bands for all patients (columns). Edges from the interaction matrices that have corresponding direct anatomical connections are marked by 'o' and their sizes encode the number of estimated streamlines between each pair of regions. The functional edges without any corresponding anatomical connections are marked by '.'. All edges are also color-coded based on the Euclidean distance between the nodes (i.e., brain region pairs). each red line represents the linear fit to the scatter plot and each dashed red lines represent the 95% confidence interval. The slope (mean across patients = 0.64 ± 0.14), the small intercept (mean = 0.02 ± 0.01), and relatively moderate R^2 values (mean = 0.52 ± 0.05) of the linear fit to the points highlights the large similarity between the interaction matrices of one frequency band and the interaction matrices of another frequency band. These plots also demonstrate that the high values of the interaction matrix elements are highly predictive of the presence of anatomical connectivity between brain regions. ***(b)*** Each point in the scatter plot represents the functional connectivity value estimated from the binarized band-passed power amplitude states correlations (i.e., co-activation rates) at the lowest (i.e., θ) and highest (i.e., high γ) frequency bands for all patients. Compared the results in panel ***(a)***, the relationship between the correlations values at low- and high-frequency bands is less strong, as highlighted by the poor fit of the linear fits across all patients (mean slope = 0.56 ± 0.14, mean intercept = 0.34 ± 0.11, and mean $R^2 = 0.21 \pm 0.06$). Note that, although at low frequencies (i.e., θ) the correlation values are distributed in a manner that is comparable to that of the interaction matrices in ***a***, at higher frequencies the correlation values between the more distant electrodes is relatively high and provides less differentiation of direct anatomical connectivity.

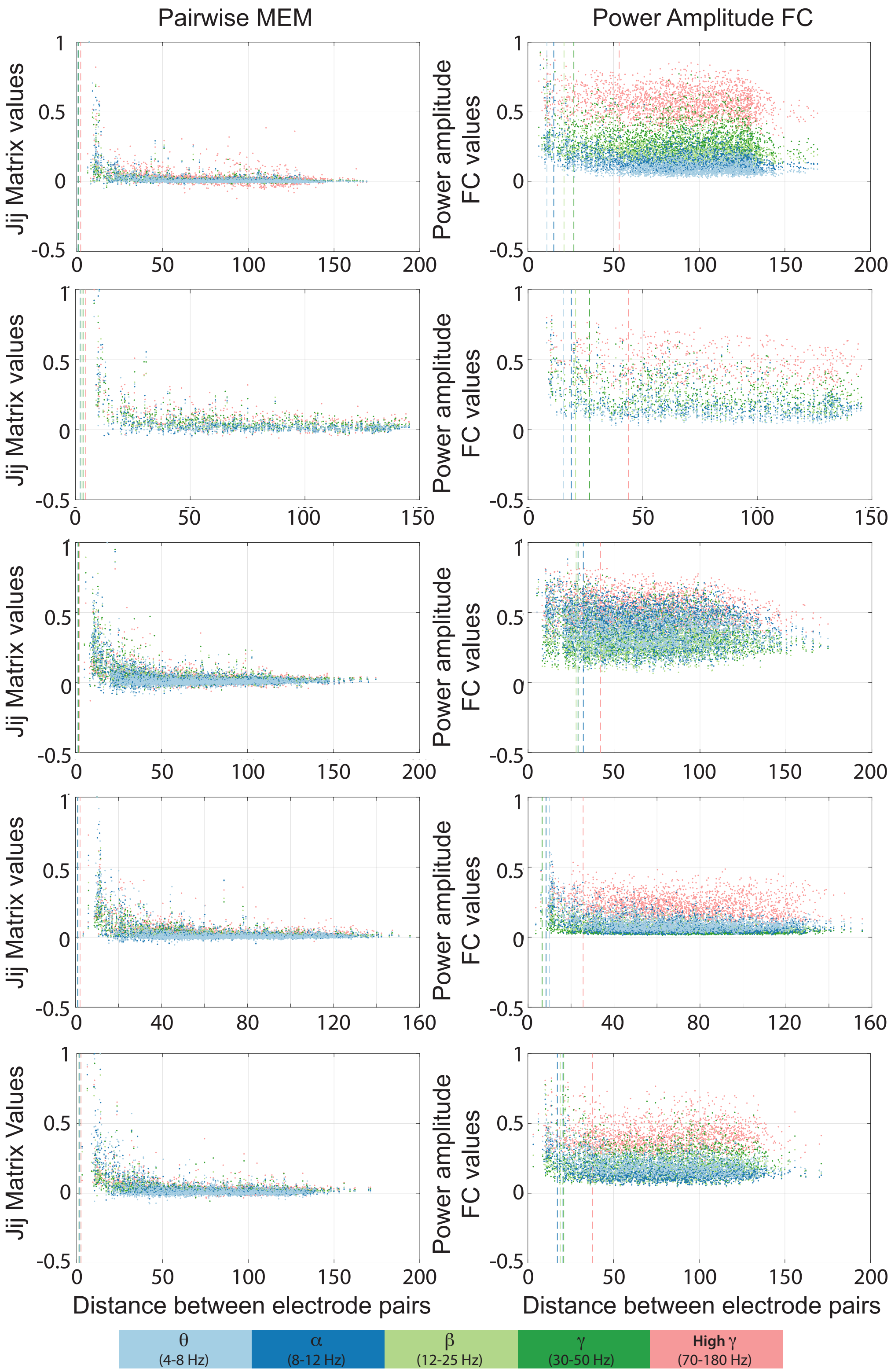

Figure 4. Dependences of maximum entropy interactions and pairwise correlations on inter-electrode distance. (Left) We plot the normalized maximum entropy interactions J_{ij} against the Euclidian distance between the corresponding electrodes i and j, using referential montage and binarization threshold of zero. (Right) Similarly, we plot the normalized pairwise correlations in binarized power amplitude states (i.e., co-activation rates) against the corresponding inter-electrode distances. Data are color-coded based on the frequency band. The dashed vertical lines indicate the average J_{ij} and pairwise correlation values for each frequency band, weighted by the Euclidean distances between electrodes. Distance-weighted average values indicate that pairwise correlation values become progressively more skewed toward nearby electrodes from higher to lower frequency bands (two-sided Wilcoxon rank-sum test, $p < 0.05$, FDR corrected for multiple comparisons across all possible frequency pairs). However, the distributions of distance weighted average J_{ij} are very similar across all bands and all are similarly skewed towards nearby electrodes.

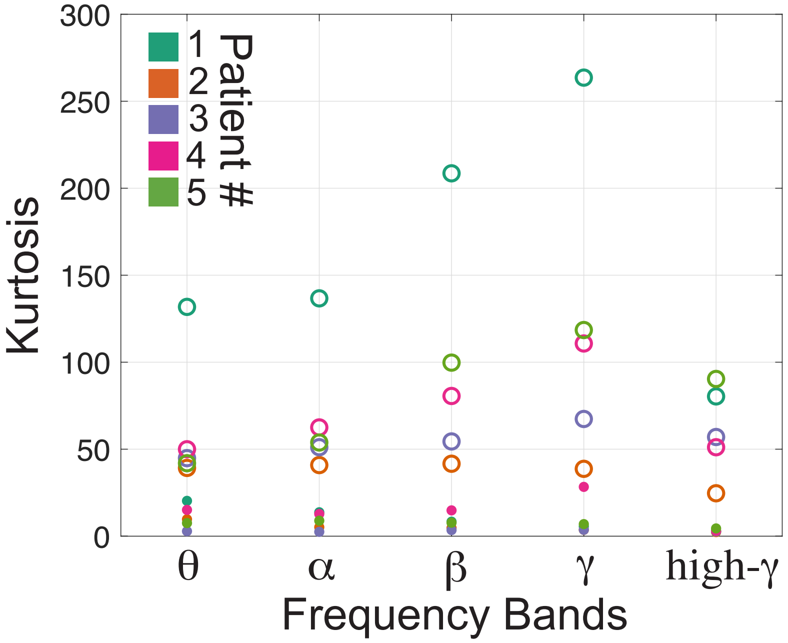

Figure 5. Kurtosis of J_{ij} interaction weights and pairwise correlation distributions. The color-coded 'o' and '.' markers indicate the kurtosis of J_{ij} interaction weights and pairwise correlation distributions for all patients and frequency bands. Statistical comparisons reveal that the kurtosis values calculated from J_{ij} interaction weights are significantly (paired $t-$test, $p < 0.05$, $p = 6.4 \times 10^{-7}$, $N = 5$ patients $\times$ 5 frequency bands) higher than those of correlation value distributions.

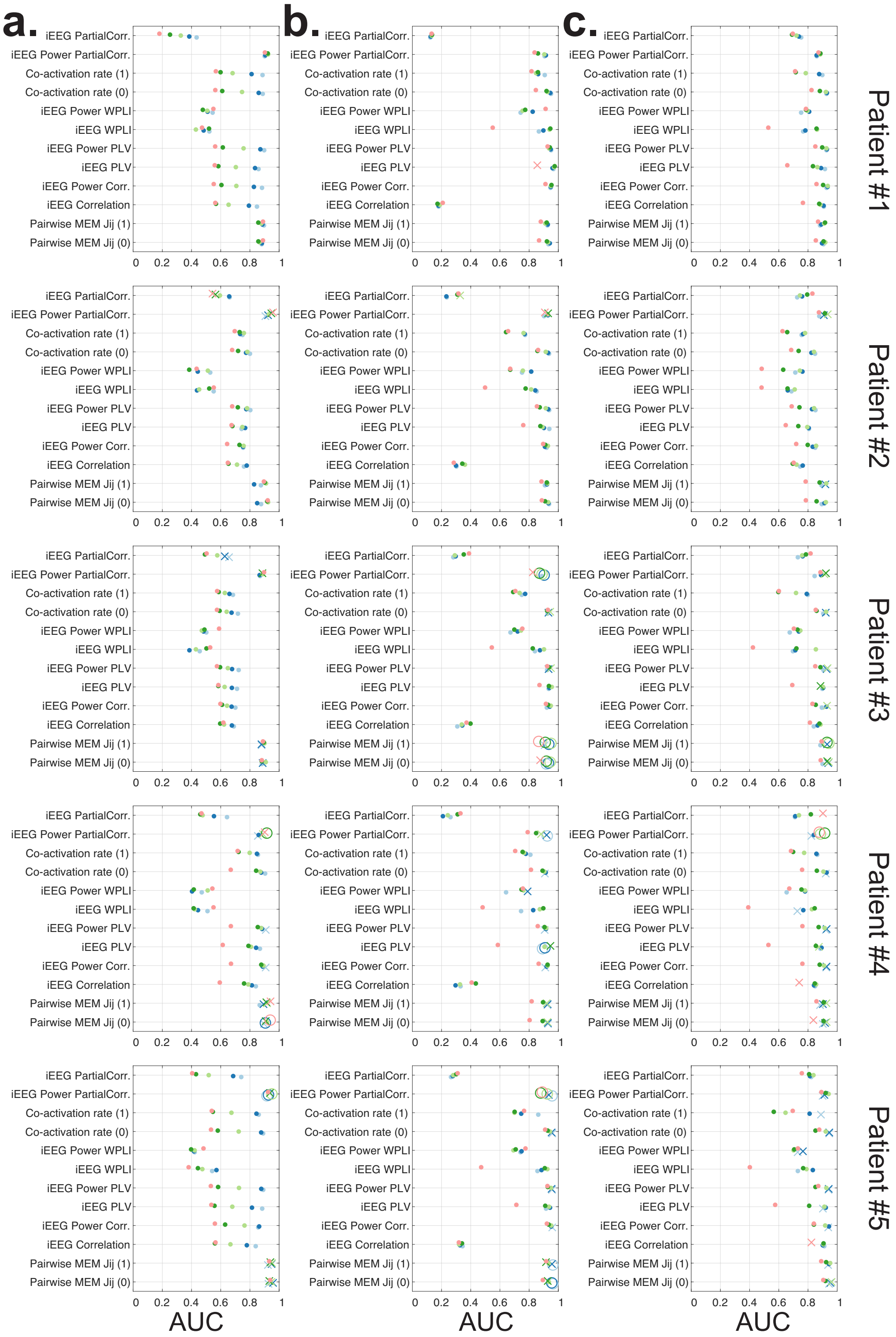

Figure 6. Predicting structural connectivity (binarized) from functional connectivity estimates. Plots display the detection accuracy (measure via AUC) of structural connectivity between brain regions using several functional connectivity estimation methods, for all patients (rows) and three recording montages – referential ***(a)***, local multiple linear regression ***(b)***, and global mean signal regression ***(c)*** montages. The functional connectivity methods include, pairwise MEM and correlation calculated from power amplitude states binarized at 0 and 1, Pearson correlation, partial correlation, PLV, and WPLI calculated from iEEG and their band-passed power time series. The results for different frequency bands are color-coded based on the legend in panel ***e*** of manuscript Figure 6. The AUC values significantly higher than those of geometric structural nulls (one-sided permutation test, $n = 10{,}000$, $p < 0.05$) are depicted as 'X', and AUC values significant after FDR correction for multiple comparisons across frequency bands are depicted as 'O'.

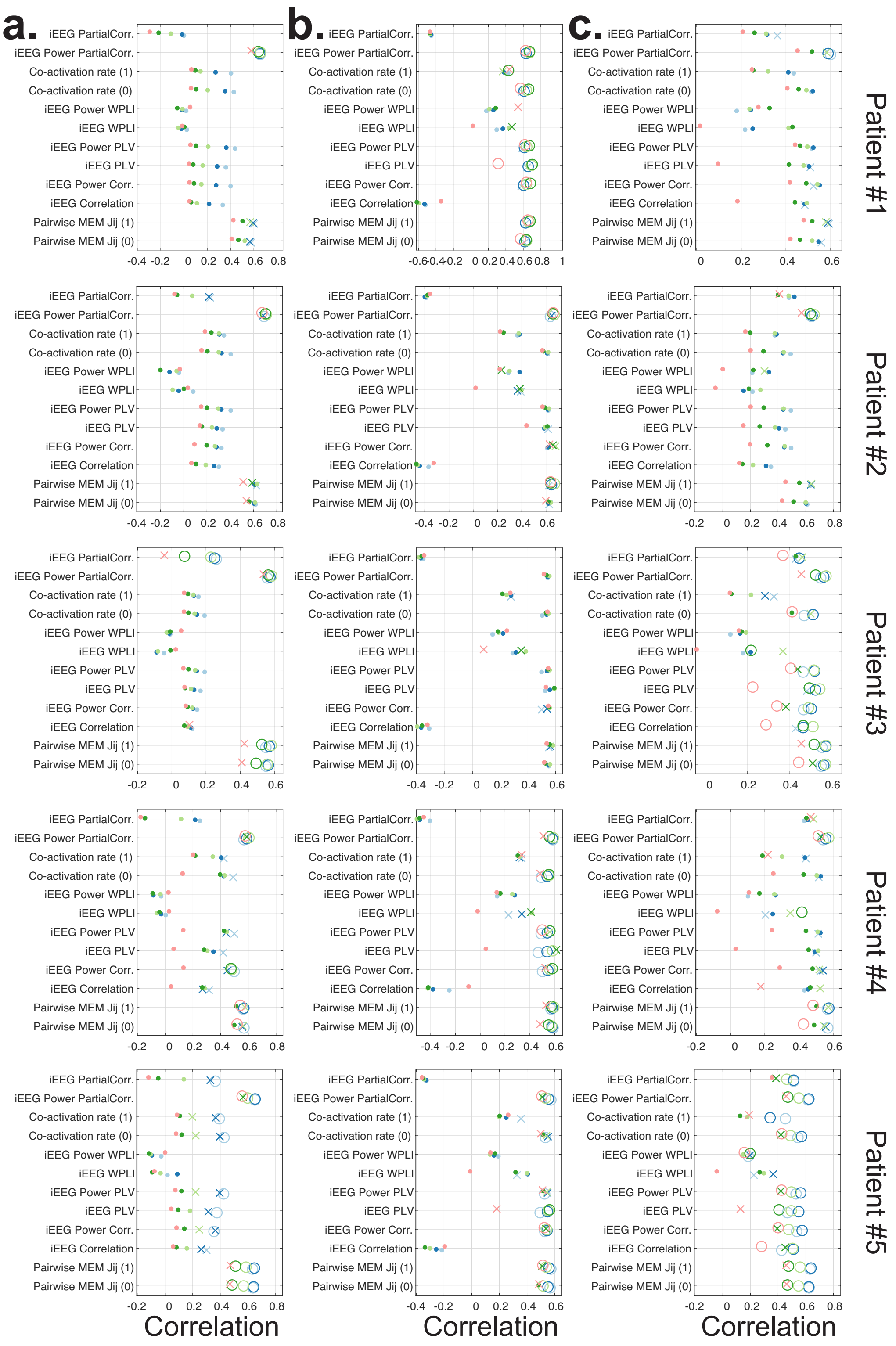

Figure 7. Coupling between structural (log normalized streamline counts) and functional connectivity estimates. Plots display the structure-function coupling (measured as the correlation between structural and functional connectivity matrices) of sampled brain regions using several functional connectivity estimation methods, for all patients (rows) and three recording montages – referential ***(a)***, local multiple linear regression ***(b)***, and global mean signal regression ***(c)*** montages. The functional connectivity methods include, pairwise MEM and correlation calculated from power amplitude states binarized at 0 and 1, Pearson correlation, partial correlation, PLV, and WPLI calculated from iEEG and their band-passed power time series. The results for different frequency bands are color-coded based on the legend in panel ***e*** of manuscript Figure 6. The correlation values significantly higher than those of geometric structural nulls (one-sided permutation test, n = 10,000, $p < 0.05$) are depicted as 'X', and values significant after FDR correction for multiple comparisons across frequency bands are depicted as 'O'.

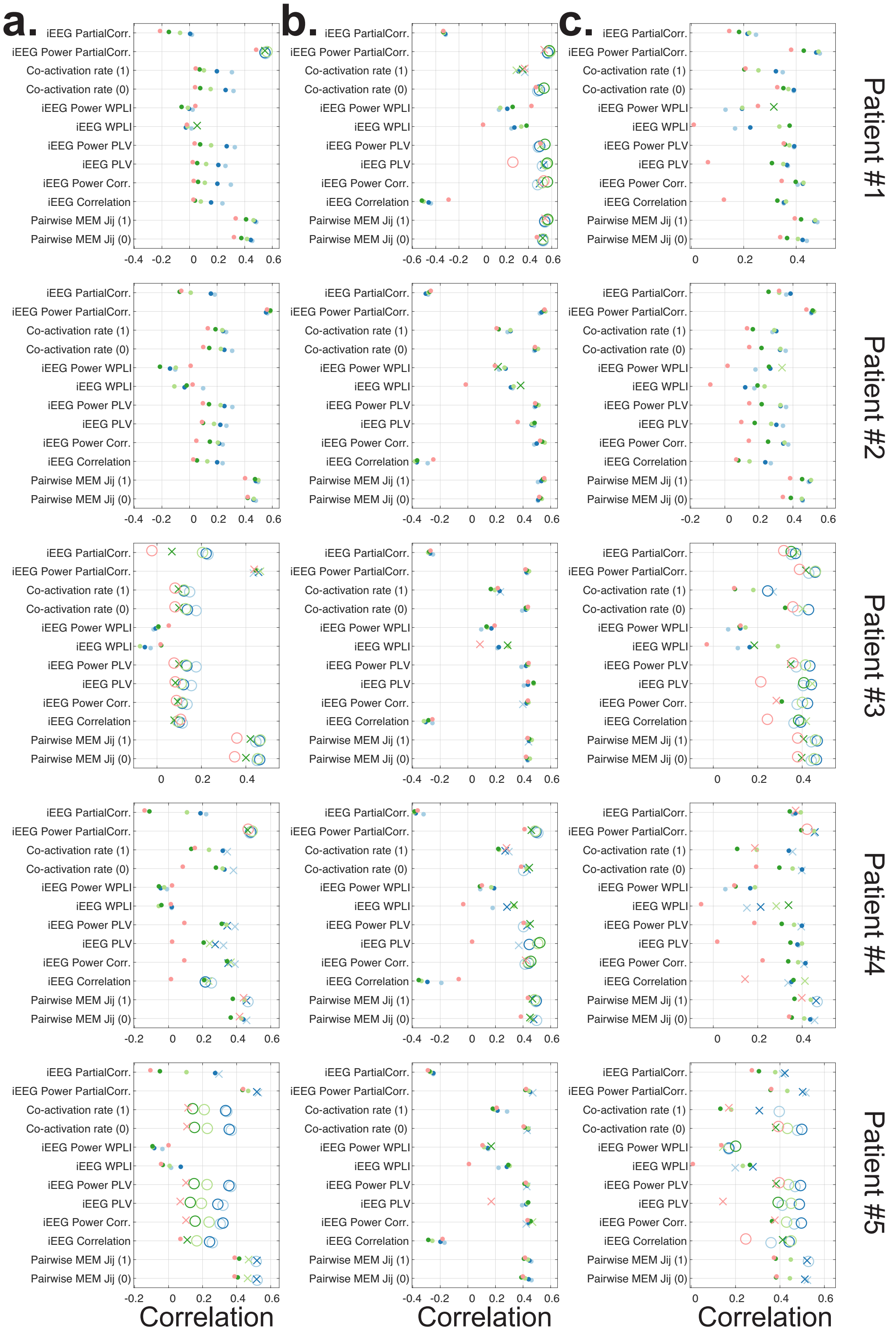

Figure 8. Coupling between structural (streamline counts) and functional connectivity estimates. Plots display the structure-function coupling (measure as the correlation between structural and functional connectivity matrices) of sampled brain regions using several functional connectivity estimation methods, for all patients (rows) and three recording montages – referential ***(a)***, local multiple linear regression ***(b)***, and global mean signal regression ***(c)*** montages. The functional connectivity methods include, pairwise MEM and correlation calculated from power amplitude states binarized at 0 and 1, Pearson correlation, partial correlation, PLV, and WPLI calculated from iEEG and their band-passed power time series. The results for different frequency bands are color-coded based on the legend in panel ***e*** of manuscript Figure 6. The correlation values significantly higher than those of geometric structural nulls (one-sided permutation test, n = 10,000, $p < 0.05$) are depicted as 'X', and values significant after FDR correction for multiple comparisons across frequency bands are depicted as 'O'. Note that although the results are comparable to Supplementary Figure 7, log normalization of streamline counts results in overall higher structure-function similarities.

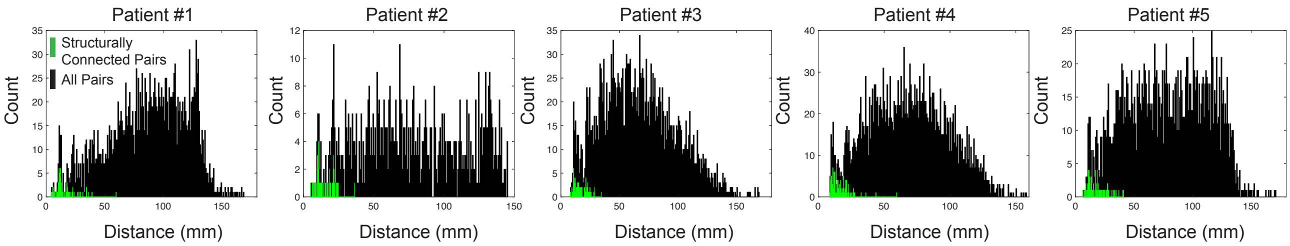

Figure 9. Distance between iEEG electrodes. Plots show the distributions (black) of the Euclidean distances between all electrode pairs for all five patients. The distributions (green) of the distances between all electrode pairs recording from structurally connected regions are also presented. Note that the majority of the structural connections are short-ranged between regions covered by iEEG electrodes.

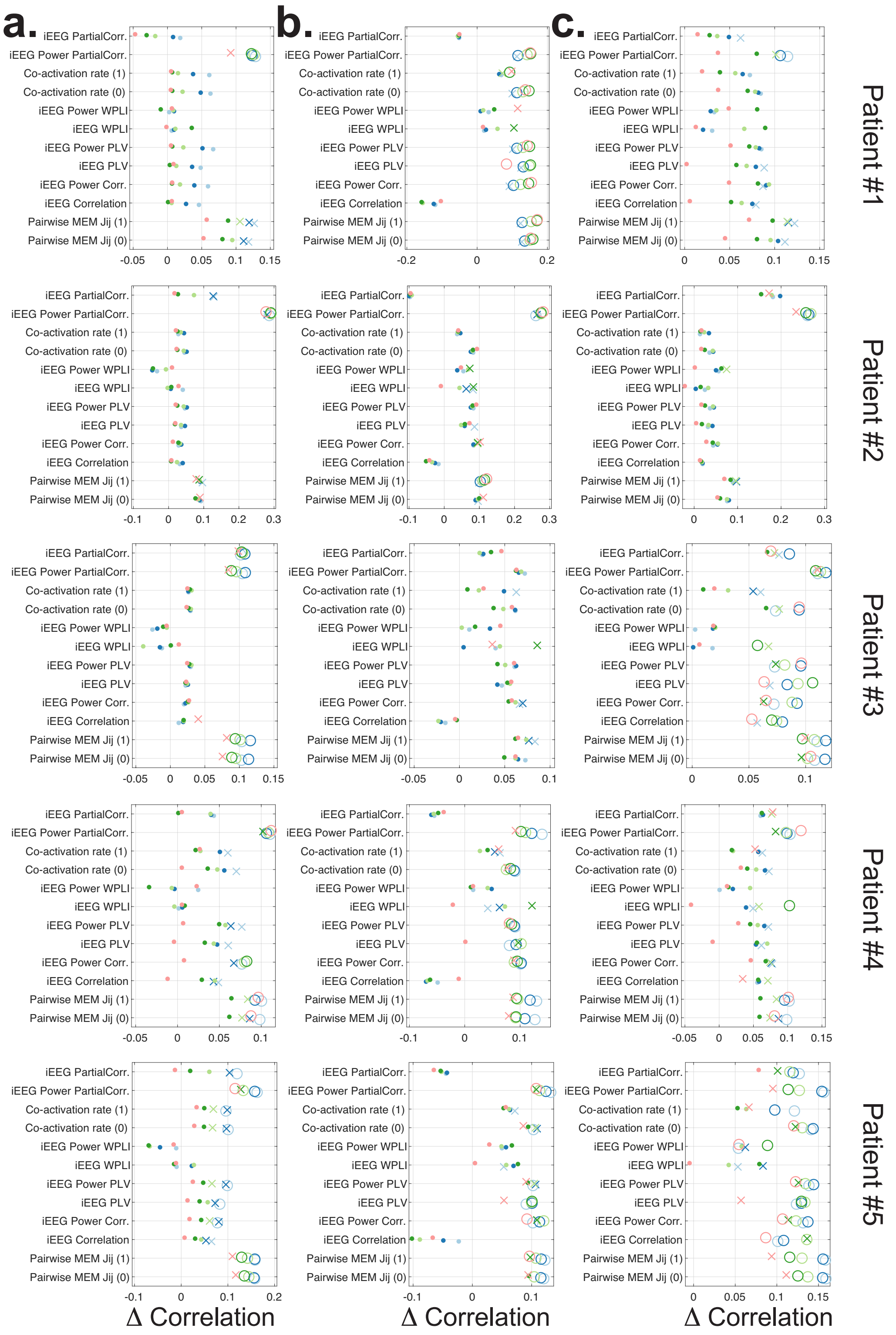

Figure 10. Similarity between structural (log normalized streamline count) and functional connectivity estimates. Plots display the difference between the structure-function coupling (measured as the correlation between structural and functional connectivity matrices) of sampled brain regions and the average coupling values calculated from structural null matrices using several functional connectivity estimation methods, for all patients (rows) and three recording montages – referential ***(a)*** , local multiple linear regression ***(b)***, and global mean signal regression ***(c)*** montages. The functional connectivity methods include, pairwise MEM and correlation (i.e., co-activation rates) calculated from power amplitude states binarized at 0 and 1, Pearson correlation, partial correlation, PLV, and WPLI calculated from iEEG and their band-passed power time series. The results for different frequency bands are color-coded based on the legend in panel ***e*** of manuscript Figure 6. The correlation values significantly higher than those of geometric structural nulls (one-sided permutation test, n = 10,000, $p < 0.05$) are depicted as 'X', and values significant after FDR correction for multiple comparisons across frequency bands are depicted as 'O'.

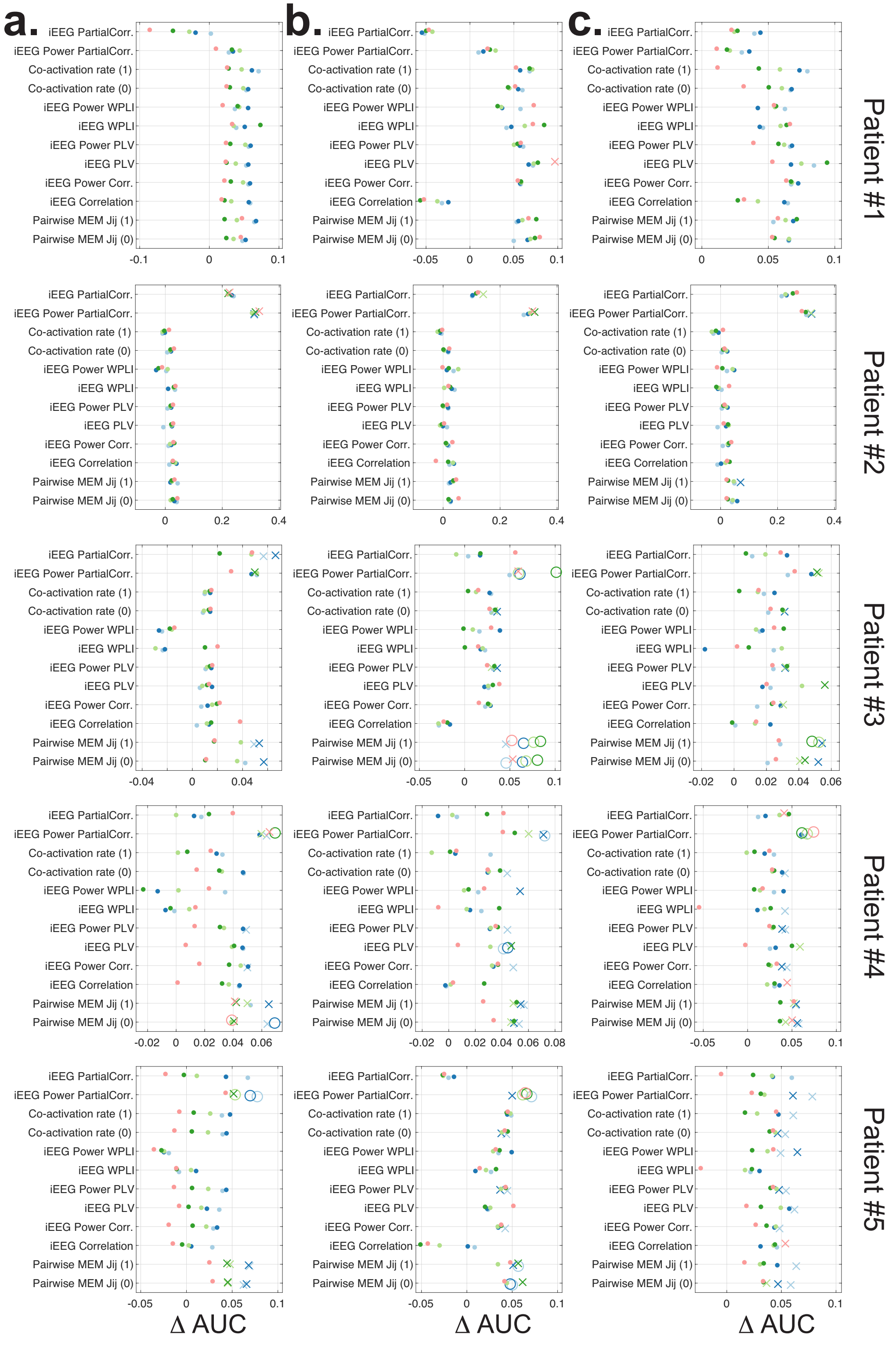

Figure 11. Predicting structural connectivity (binarized) from functional connectivity estimates. Plots display the difference between the detection accuracy (measured via AUC) of structural connectivity between brain regions and the average detection accuracy values calculated from structural null matrices using several functional connectivity estimation methods, for all patients (rows) and three recording montages – referential ***(a)***, local multiple linear regression ***(b)***, and global mean signal regression ***(c)*** montages. The functional connectivity methods include, pairwise MEM and correlation (i.e., co-activation rates) calculated from power amplitude states binarized at 0 and 1, Pearson correlation, partial correlation, PLV, and WPLI calculated from iEEG and their band-passed power time series. The results for different frequency bands are color-coded based on the legend in panel ***e*** of manuscript Figure 6. The AUC values significantly higher than those of geometric structural nulls (one-sided permutation test, $n = 10{,}000$, $p < 0.05$) are depicted as 'X', and values significant after FDR correction for multiple comparisons across frequency bands are depicted as 'O'.

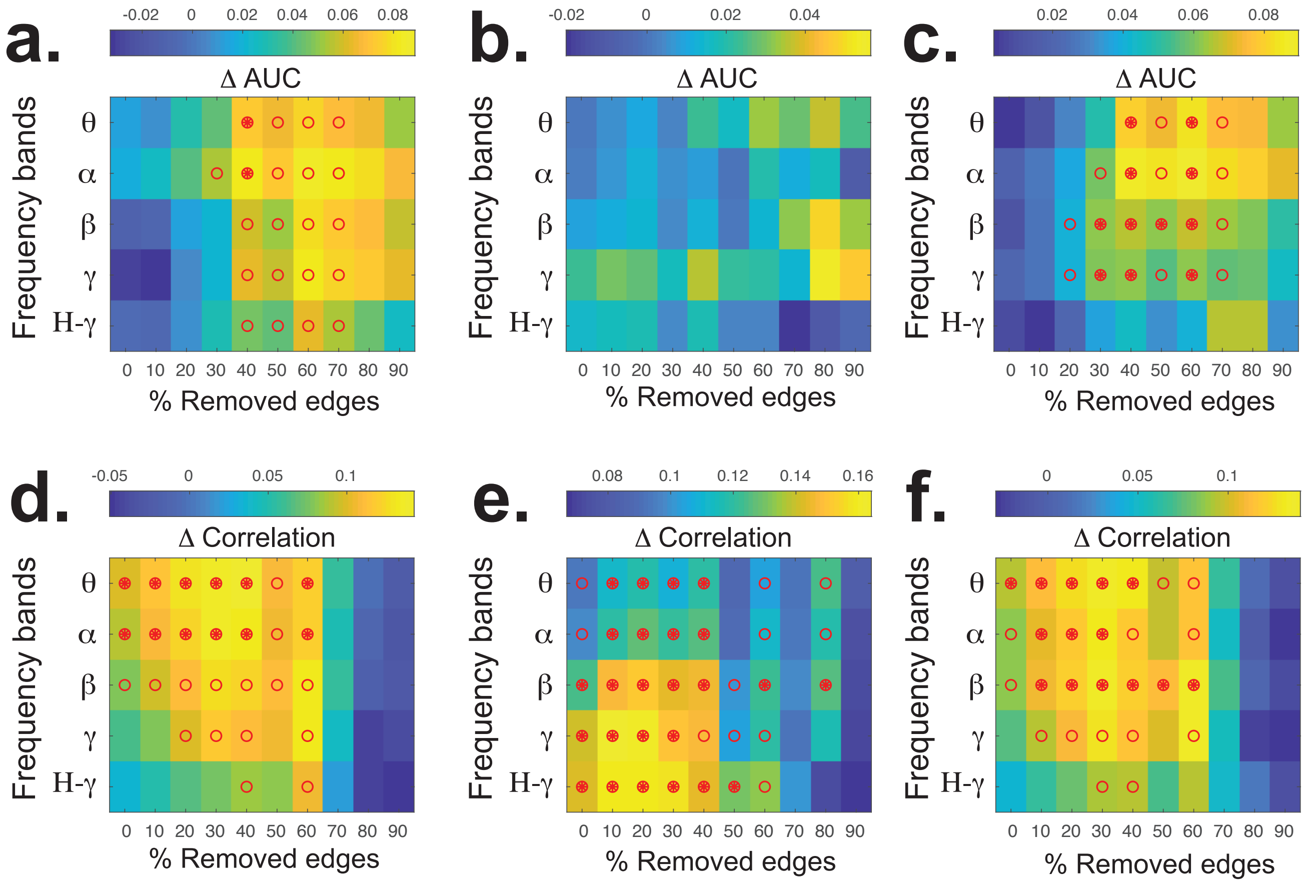

Figure 12. **Removing weak structural connections increases the structure-function coupling in patient #1.** Top plots display the difference between the detection accuracy (i.e., area under the ROC Curve (AUC)) of structural connections between brain regions using pairwise MEM (power amplitude states binarized at 1), and the detection accuracy of structural nulls, after removing different percentages of weak (i.e., fewer streamlines) structural edges for patient #1. We used referential ***(a)***, local multiple linear regression ***(b)***, and global mean signal regression ***(c)*** montages for results in panels ***(a-c)***, respectively. Bottom plots display the difference between the structure-function coupling (measured as the correlation between structural and functional connectivity matrices) of sampled brain regions and the average coupling values calculated from structural null matrices using pairwise MEM removing weaker structural edges, for the same patient using the same montages as ***(a-c)*** in ***(d-f)*** plots, respectively. Red markers indicate significantly high structure-function couplings, beyond that of geometric structural nulls (one-sided permutation test, $n = 10,000$, $p < 0.05$ marked with 'open' circles, and FDR corrected for multiple comparisons across all frequency bands marked with 'closed' circles). These results show that removing around $20\% - 60\%$ of weaker edges increase the structure-function coupling in our sample patient #1.

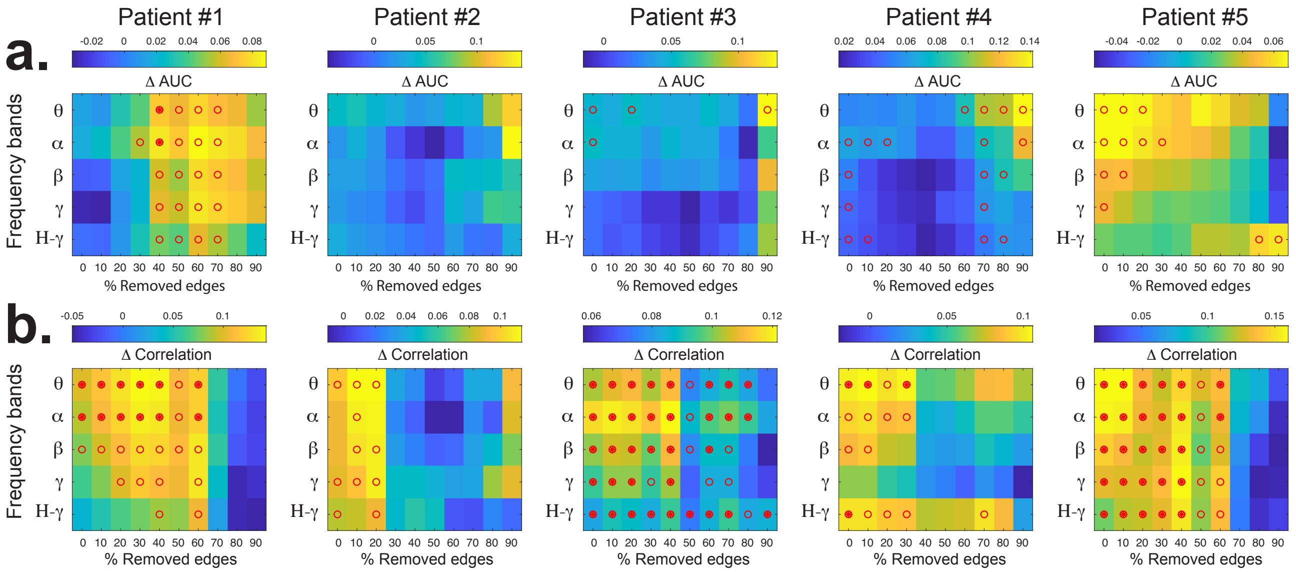

Figure 13. Removing weak structural connections effects structure-function coupling differently across patients. ***(a)*** Plots display the difference between the detection accuracy (i.e., area under the ROC Curve (AUC)) of structural connections between brain regions using pairwise MEM (power amplitude states binarized at 1), and the detection accuracy of structural nulls, after removing different percentages of weak (i.e., fewer streamlines) structural edges for all patients using referential montage. ***(b)*** Plots display the difference between the structure-function coupling (measured as the correlation between structural and functional connectivity matrices) of sampled brain regions and the average coupling values calculated from structural null matrices using pairwise MEM removing weaker structural edges, for all patients using referential montage. Red markers indicate significantly high structure-function couplings, beyond that of geometric structural nulls (one-sided permutation test, $n = 10,000$, $p < 0.05$ marked with 'open' circles, and FDR corrected for multiple comparisons across all frequency bands marked with 'closed' circles). Note that the effect of removing weaker edges on the structure-function coupling is heterogeneous across patients. Nevertheless, removing weaker edges overall increased the correlation between the two modalities.

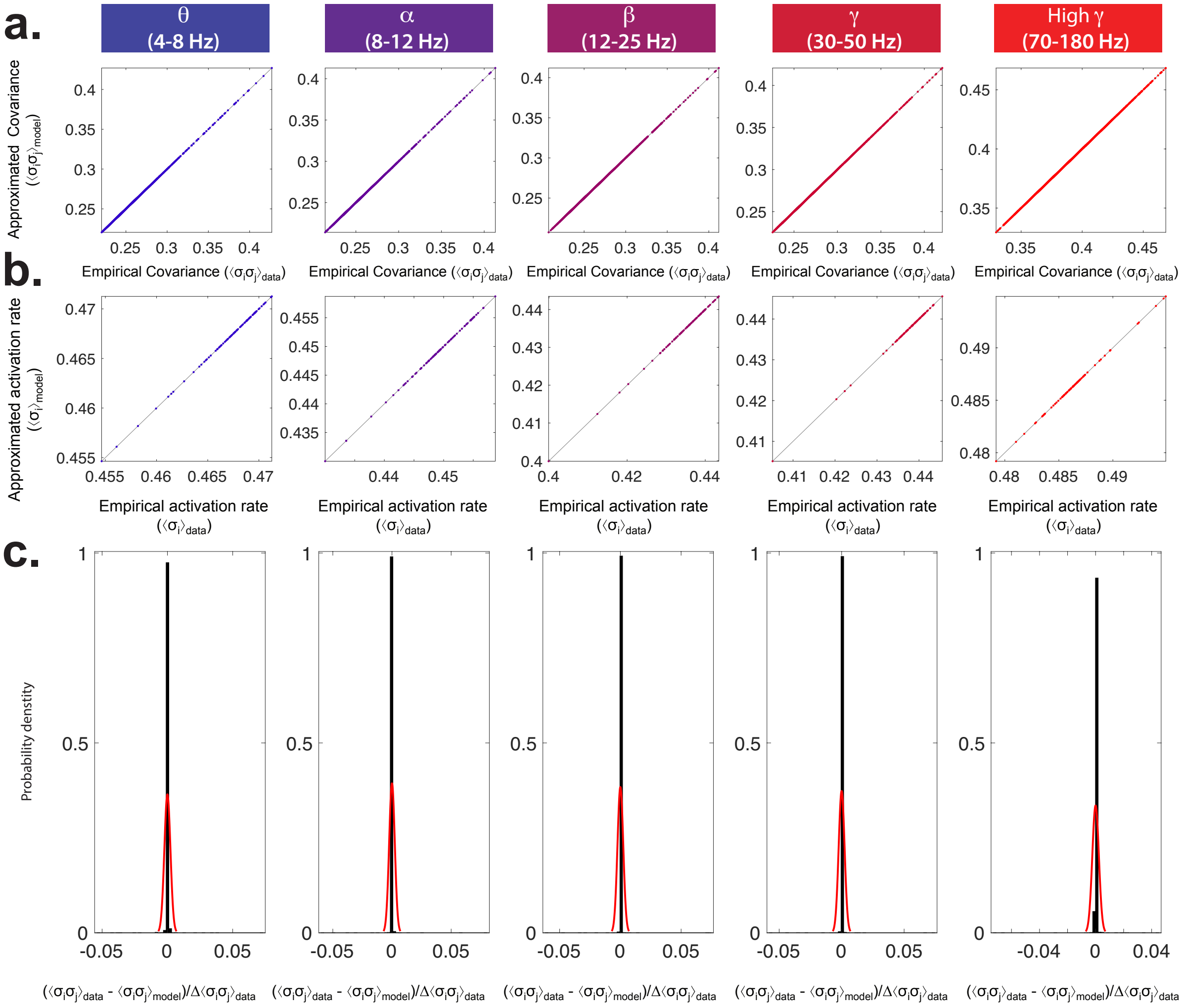

Figure 14. Accuracy of the maximum entropy model in reconstructing the empirical covariance structure using pseudo-likelihood maximization scheme *(a)* Approximated pairwise correlations ($< \sigma_i \sigma_j >_{model}$) under the maximum entropy model versus the observed correlations ($< \sigma_i \sigma_j >_{data}$) from a sample patient (Patient #1) over 13 hours of inter-ictal recordings. ***(b)*** Approximated activation rate ($< \sigma_i \sigma_j >_{model}$) under the maximum entropy model versus the observed correlations ($< \sigma_i \sigma_j >_{data}$) from the same patient. ***(c)*** Distribution of the differences between the true and model pairwise correlations, normalized by the error in the data $\Delta < \sigma_i \sigma_j >$. For reference, the red line is a Gaussian fit to the distribution. The small mean ($< 10^{-6}$ across all patients) and standard deviation (< 0.002 across all patients) of the Gaussian fit demonstrates that the learning algorithm reconstructs the pairwise correlations well within experimental precision.

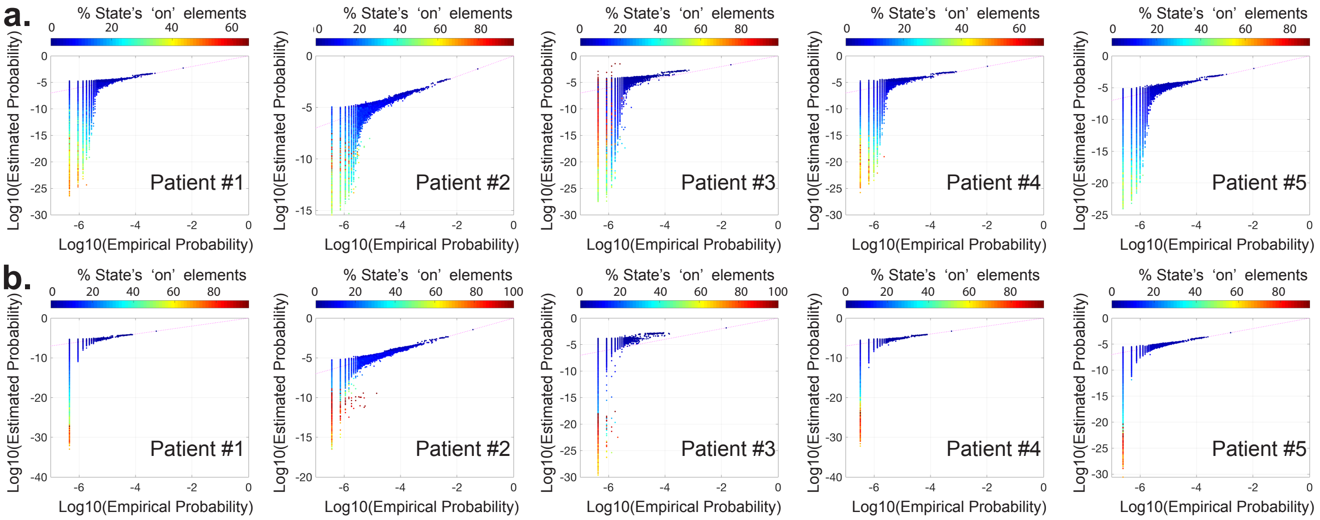

Figure 15. Accuracy of the maximum entropy model in predicting activity patterns. Plots represent the empirical and estimated probabilities of all empirically observed states for all patients at θ ***(a)***, and High γ ***(b)*** bands using local multiple linear regression montage and state binarization threshold of '1'. The *partition function* (i.e., the normalization constant in the Boltzmann distribution) was approximated from the empirical probability of the *silent* state (see [1;2] for more details). The co-activation states' size (i.e., the percentage 'on' elements of states) is color-coded based on the color bar on top of each panel.

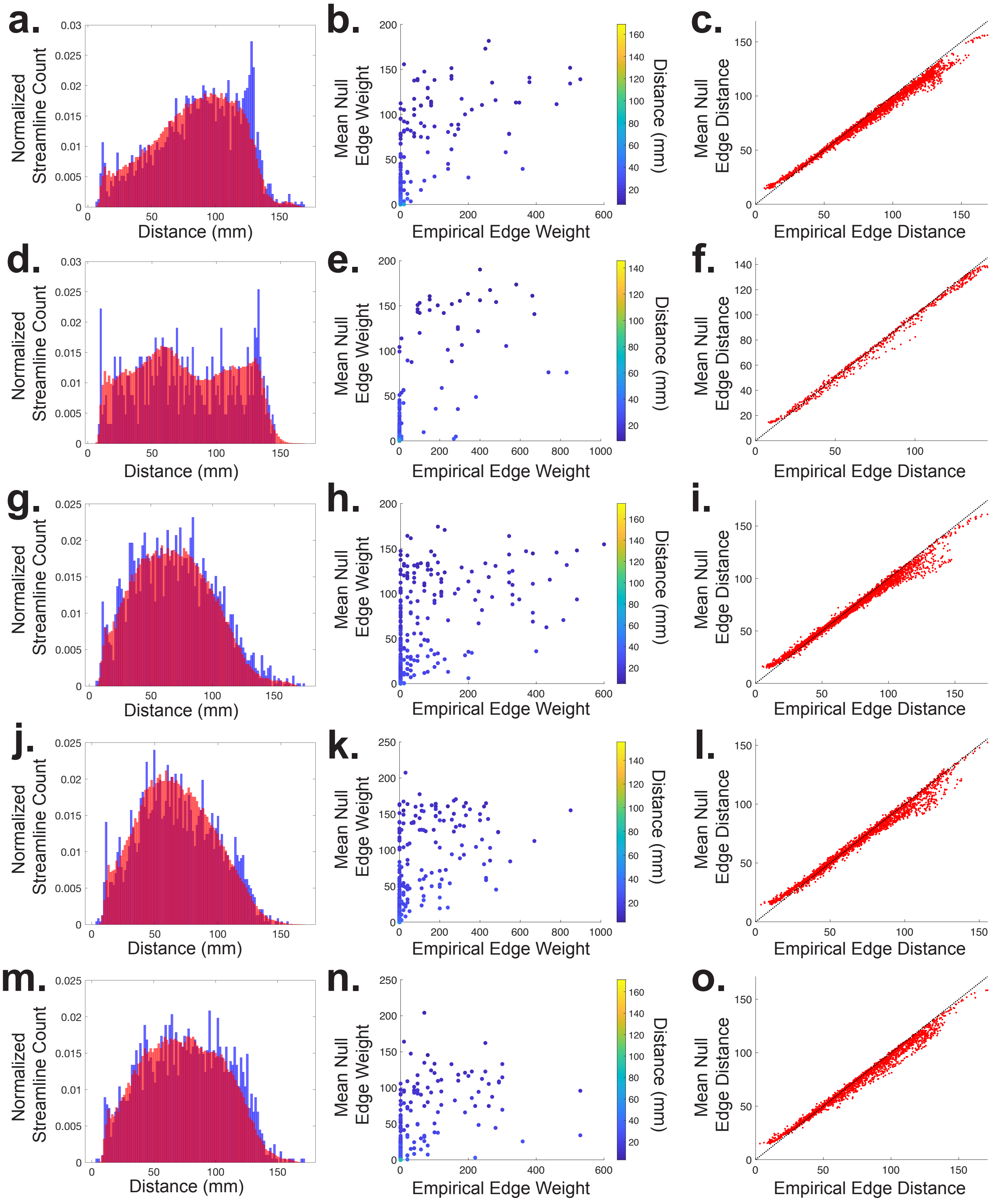

Figure 16. Geometric structural connectivity nulls. Rows 1 to 5 display results for patients #1 to #5, top to bottom, respectively. ***(a,d,g,j,m)*** Histograms represent the empirical (blue) and the null (red) normalized number of the streamlines based on the distance between pairs of connected brain regions (i.e., electrodes). ***(b,e,h,k,n)*** Scatter plots represent the empirical and the mean number of null streamlines for each structural edge. ***(c,f,i,l,o)*** Scatter plots represent the empirical distance between electrodes (mm) and the mean null distance between all possible pairs of sampled brain regions.

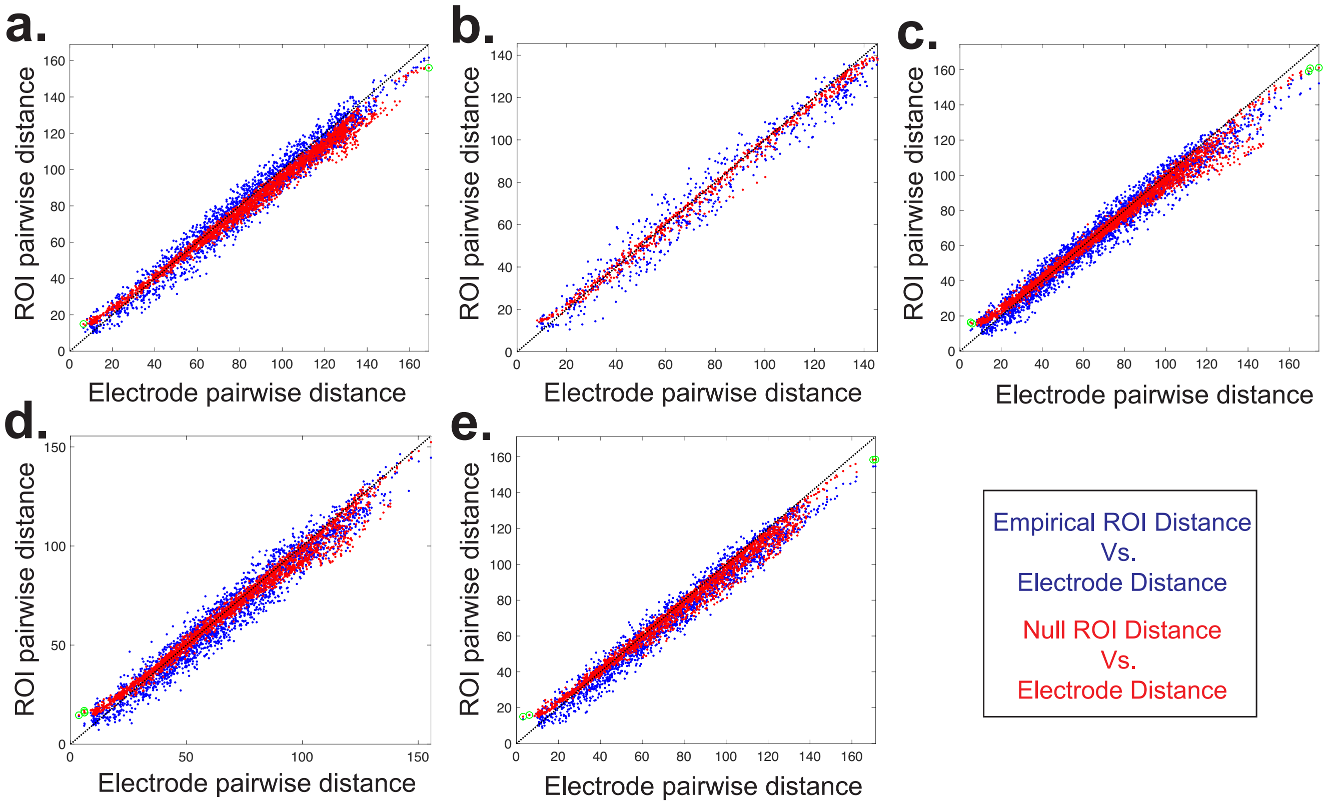

Figure 17. Pairwise distance between iEEG electrodes, empirically selected ROIs, and null ROIs. ***(a-e)*** Scatter plots represent the empirical Euclidean distance between electrodes (mm) on the x-axis, and empirical distance between selected brain regions' centers (blue), as well as the mean distance between null brain regions' centers (red) on the y-axis, for patients #1 to #5 respectively. Note that across all patients, the empirical distance between iEEG electrode pairs is very similar to the average distance between pairs of resampled null brain regions. Green circles highlight the few pairs of electrodes (average of ∼3 pairs per patient) with significantly (one-sided permutation test, $n = 10,000$, $p < 0.05$, FDR corrected for multiple comparisons across all possible pairs) different pairwise distances than those observed in their corresponding null brain region pairs. Overall, the resampling distance error of null regions is significantly smaller than the error associated with the physical distance between iEEG electrodes and the centroids of the most proximate ROIs (see Supplementary Figure 18 for details).

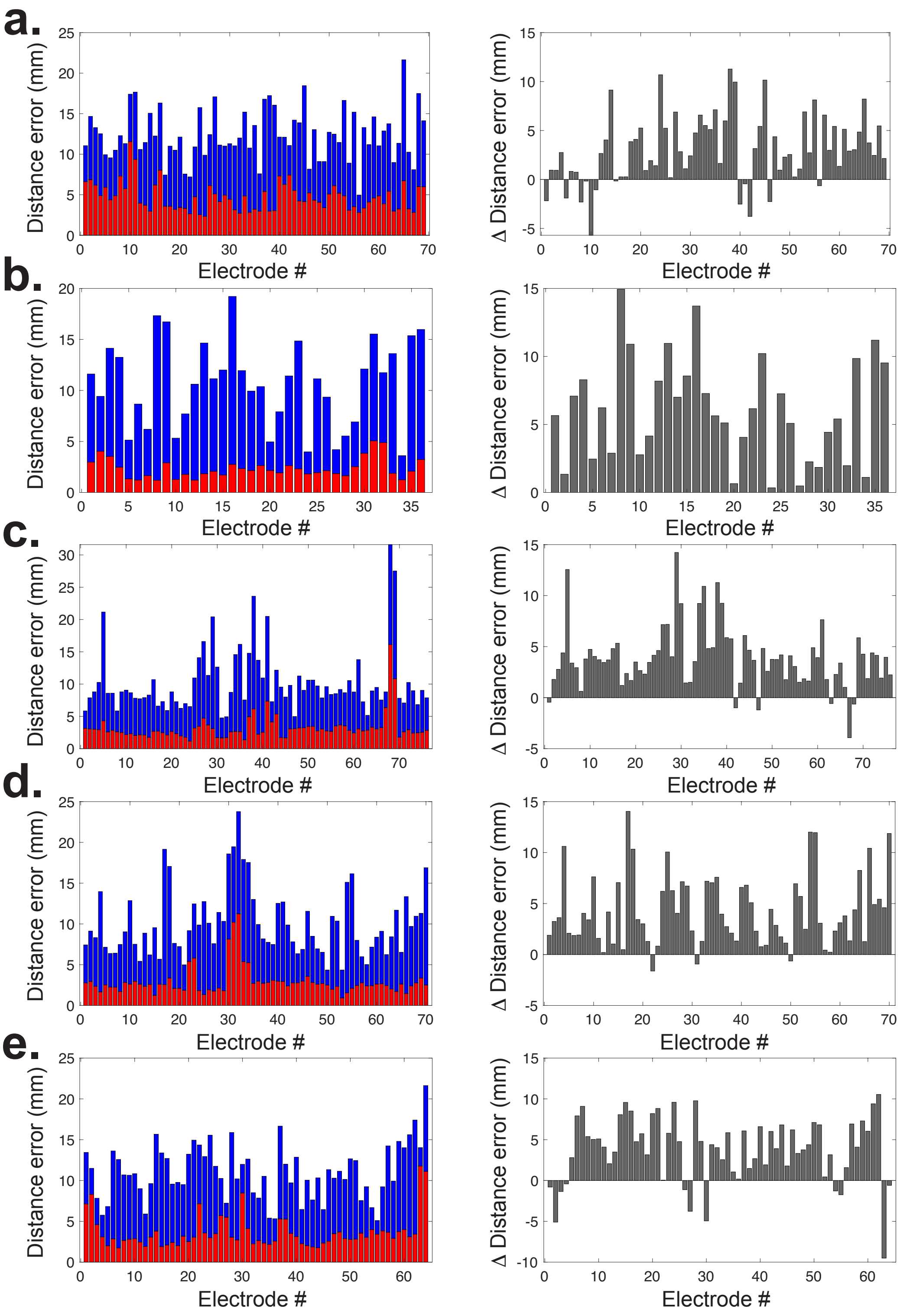

Figure 18. Average distance error between null ROI pairs versus distance between electrode and centers of empirical ROIs. The stacked bar plots on left shows the distance between the electrode and the corresponding closest ROIs in blue, and the average error in distance between each resampled null ROI and the rest of null ROIs in red. The bar plots on the right shows the difference between the two blue and red bars from the left plots. Results from each patient are presented at each row ***(a-e)***. We note that the empirical distance between the electrode and the ROI center is significantly larger than the average null distance error across all patients (two-sided t−test, $p = 9.73 \times 10^{-12}$, $p = 3.16 \times 10^{-13}$, $p = 9.65 \times 10^{-14}$, $p = 2.6 \times 10^{-16}$, $p = 3.29 \times 10^{-13}$). Together, these results demonstrate that the distance error of the geometric null is smaller than the error associated with selecting ROIs closest to the iEEG electrodes, which suggests that the null is sufficiently conservative.

References

1. Ganmor, E., Segev, R. & Schneidman, E. Sparse low-order interaction network underlies a highly correlated and learnable neural population code. *Proceedings of the National Academy of Sciences* **108**, 9679–9684 (2011).

2. Tkačik, G. *et al.* Searching for collective behavior in a large network of sensory neurons. *PLoS computational biology* **10**, e1003408 (2014).
